# Structural analysis of the basal state of the Artemis:DNA-PKcs complex

**DOI:** 10.1093/nar/gkac564

**Published:** 2022-07-08

**Authors:** Go Watanabe, Michael R Lieber, Dewight R Williams

**Affiliations:** Department of Pathology, Department of Biochemistry & Molecular Biology, Department of Molecular Microbiology & Immunology, and Section of Computational & Molecular Biology, USC Norris Comprehensive Cancer Center, University of Southern California Keck School of Medicine, 1441 Eastlake Ave, Rm. 5428, Los Angeles, CA 90089, USA; Department of Pathology, Department of Biochemistry & Molecular Biology, Department of Molecular Microbiology & Immunology, and Section of Computational & Molecular Biology, USC Norris Comprehensive Cancer Center, University of Southern California Keck School of Medicine, 1441 Eastlake Ave, Rm. 5428, Los Angeles, CA 90089, USA; Eyring Materials Center, John Cowley Center for High Resolution Electron Microscopy, Arizona State University, Tempe, AZ 85281, USA

## Abstract

Artemis nuclease and DNA-dependent protein kinase catalytic subunit (DNA-PKcs) are key components in nonhomologous DNA end joining (NHEJ), the major repair mechanism for double-strand DNA breaks. Artemis activation by DNA-PKcs resolves hairpin DNA ends formed during V(D)J recombination. Artemis deficiency disrupts development of adaptive immunity and leads to radiosensitive T- B- severe combined immunodeficiency (RS-SCID). An activated state of Artemis in complex with DNA-PK was solved by cryo-EM recently, which showed Artemis bound to the DNA. Here, we report that the pre-activated form (basal state) of the Artemis:DNA-PKcs complex is stable on an agarose-acrylamide gel system, and suitable for cryo-EM structural analysis. Structures show that the Artemis catalytic domain is dynamically positioned externally to DNA-PKcs prior to ABCDE autophosphorylation and show how both the catalytic and regulatory domains of Artemis interact with the N-HEAT and FAT domains of DNA-PKcs. We define a mutually exclusive binding site for Artemis and XRCC4 on DNA-PKcs and show that an XRCC4 peptide disrupts the Artemis:DNA-PKcs complex. All of the findings are useful in explaining how a hypomorphic L3062R missense mutation of DNA-PKcs could lead to insufficient Artemis activation, hence RS-SCID. Our results provide various target site candidates to design disruptors for Artemis:DNA-PKcs complex formation.

## Graphical Abstract

**Graphical Abstract:**
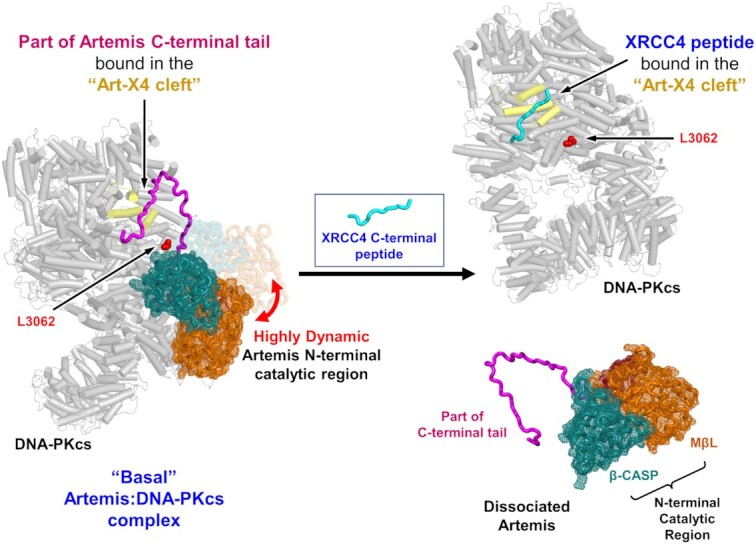
A basal state of Artemis:DNA-PKcs structure reveals that the Artemis catalytic region is dynamically positioned externally to DNA-PKcs. An XRCC4 peptide disrupts the Artemis:DNA-PKcs complex at a critical Art-X4 cleft.

## INTRODUCTION

Nonhomologous DNA end joining (NHEJ) is the primary pathway by which DNA double-strand breaks (DSBs) are repaired in vertebrate cells (1). Dividing cells incur ∼10 DSBs per cell per day (2). These are the most damaging DNA lesions, as failure to resolve DSBs causes the loss of extensive portions of chromosomes. While homology-directed repair of DSBs depends on the presence of a homology donor, and therefore primarily proceeds during S and G2 phases, NHEJ occurs throughout the cell cycle and is therefore utilized for the repair of most DSBs and essentially all DSBs during unscheduled DNA synthesis (2).

NHEJ is initiated at DSBs by the Ku70/80 heterodimer binding at the break site, which then recruits core NHEJ factors. DNA-dependent protein kinase catalytic subunit (DNA-PKcs), an important regulator of NHEJ, binds to Ku70/80 bound at dsDNA ends and undergoes autophosphorylation (3–6). Even without DNA damage, Artemis exists primarily in a complex with DNA-PKcs (7). Thus, when a DSB arises, the entire Artemis:DNA-PKcs complex likely binds to permit Artemis to function as the key endonuclease at the DNA ends (7). This nuclease complex is essential in opening the DNA hairpins generated by the RAG nuclease during V(D)J recombination. DNA polymerases μ and λ, and in the case of early lymphocytes, terminal deoxynucleotidyl transferase (TdT) are known to add nucleotides at the NHEJ junction (1). Finally, ligase IV complexes, consisting of X-ray repair cross-complementing protein 4 (XRCC4):DNA ligase IV complex (X4-LIV); XRCC4-like factor (XLF); and paralog of XRCC4 and XLF (PAXX), ligate the ends (2,8). The NHEJ pathway has the capacity to resolve a chemically and structurally diverse set of breaks, owing to the flexible and iterative nature by which NHEJ factors process DNA ends (2,9).

DNA-PKcs, a serine/threonine protein kinase, is classified as a member of the phosphoinositide 3-kinase (PI3K)-related protein kinase (PIKK) subfamily, which includes mTOR (mammalian target of rapamycin, previously known as FRAP), ATR (ataxia telangiectasia- and Rad3-related protein), ATM (ataxia telangiectasia mutated), and SMG1 (suppressor of morphogenetic effect on genitalia-1), TRRAP (transformation/transcription domain-associated protein) and DNA-PKcs (DNA-dependent protein kinase catalytic subunit) (10). Human DNA-PKcs consists of three segments, the N-term (or N-HEAT) region (1–891 aa), the Circular Cradle (or M-HEAT) region (892–2801 aa) and the Head, consisting of FAT and kinase domains (abbrev. FATKIN), (2802–4128 aa). The DNA-PKcs FAT and kinase domains are conserved in homologues across all eukaryotes as well as other PIKKs in the broader PI3K family (11). The N-HEAT and M-HEAT regions are unique to vertebrate and even invertebrate homologues of DNA-PKcs (11). However, actual DNA-dependent kinase activity, that is the dependence of such kinase activity on DNA ends, has only been demonstrated for DNA-PKcs protein from a subset of vertebrates, and not yet from invertebrates, plants or protists. It is known that there is no apparent DNA-PKcs homologue at the DNA sequence level in any model organism such as *Caenorhabditis elegans*, *Drosophila melanogaster*, *Saccharomyces cerevisiae* and *Schizosaccharomyces pombe*. Therefore, investigation of the DNA dependence of the kinase activity of homologues in other organisms will be of great interest. Similarly, any functional homologues of Artemis and the broader SNM1 family will be of interest (12).

DNA-PKcs can bind a dsDNA end both in the absence of and in the presence of Ku70/80 and activates itself by autophosphorylation (13). It has preference for S/T-Q and S/T-hydrophobic sequences for the phosphorylation of target proteins including Artemis, and there are estimated to be >60 autophosphorylation sites within DNA-PKcs (14). Functionally, the autophosphorylation has been demonstrated to occur both in *cis* for individual DNA-PKcs molecules (15) and in *trans* for some regions of DNA-PKcs (16).

Human Artemis, or SNM1C, is classified as a member of the extended structural family of the metallo-β-lactamase (MβL) fold enzymes, including SNM1A and SNM1B (17). Artemis (692 aa) consists of a catalytic domain (1–360 aa), composed of the MβL and β-CASP (CPSF-73-Artemis-SNM1-Pso2) subdomains, and an unstructured C-terminal regulatory tail (361–692 aa). The catalytic domain forms the two-metal containing active site (two Zn^2+^ ions) at the interface of the MβL and β-CASP domains (18). One active site Zn^2+^ has strong binding with H33, H35, H115 and D136 and the other Zn^2+^ (or possibly Mg^2+^) has weaker binding with D37, H38 and D136. There is also a non-active site Zn^2+^ (Cys2His2 zinc finger motif) in a peripheral location of the β-CASP domain, which is not present in SNM1A and SNM1B, which are other members of the extended structural family of MβL fold enzymes. The C-terminus is known to interact with other NHEJ components, including DNA-PKcs and DNA ligase IV (19), but more importantly is considered as a regulatory region which has been proposed to autoinhibit its endonucleolytic activity by interacting with its catalytic domain (20).

Artemis, which has intrinsic 5′ to 3′ exonuclease activity (21), acts as an endonuclease only when activated by DNA-PKcs (7). It can hydrolyze the phosphodiester bond at the junction of single-stranded to double-stranded DNA boundaries (22). At 5′ overhangs, Artemis removes the overhang intact and leaves the dsDNA blunt and with a 5′P. At 3′ overhangs, Artemis typically cuts 4 nts 3′ of the boundary to leave a 4 nt 3′overhang. Formation of the Artemis:DNA-PKcs complex and subsequent activation of Artemis endonuclease activity by DNA-PKcs is essential for hairpin DNA opening, which is necessary for the completion of V(D)J recombination during the antigen receptor development (7). Artemis deficiency leads to radiosensitive T- B- severe combined immunodeficiency (RS-SCID) (23,24).

Over two decades, a number of structural studies on DNA-PKcs have been performed using crystallography and cryo-EM (25–31). Recently, two groups reported various NHEJ synaptic complexes determined by cryo-EM. Chen *et al.* used glutaraldehyde cross-linking to stabilize two forms: a long-range synaptic complex comprising two DNA-PK complexes (each DNA end is bound by Ku70/80 and DNA-PKcs) linked by a Lig IV-XRCC4-XLF-XRCC4-Lig IV scaffold (PDB: 7LT3) and a short-range synaptic complex comprising the two DNA ends bound by Ku70/80, and linked by a Lig IV-XRCC4-XLF-XRCC4-Lig IV scaffold (PDB: 7LSY) (32). The Blundell group described two dimeric long-range synaptic complexes. One is a Ku80-mediated dimer, formed by the domain swap of the C-terminus of Ku80, (PDB: 6ZHE). The other is an XLF-mediated dimer (PDB: 7NFC), similar to the long-range synaptic complex described by Chen *et al.* (33,34).

Although a number of structural and biochemical studies on NHEJ synapsis and function have been reported, structural studies on Artemis:DNA-PKcs and the endonuclease activity have been more difficult. Structural studies with full-length Artemis have been limited because the C-terminal half of Artemis lacks any predicted structure and is likely disordered, promoting full-length Artemis to aggregate. Therefore, reported crystal structures of Artemis are limited to only a small C-terminal peptide (D485–R495) in the complex with DNA ligase IV catalytic domain (PDB: 4HTP, 3W1G) (35,36) or the N-terminal nuclease region of Artemis (37,38). Karim *et al.* reported the crystal structures of the N-terminal catalytic region of Artemis with one Zn^2+^ in the active site and one Zn^2+^ in a peripheral site (PDB: 6WO0) (37). However, DNA was not resolved despite being co-crystalized with Artemis. Later, Yosaatmadja *et al.* reported the crystal structure of a similar region but with two Zn^2+^ in the active site, along with the one in the peripheral site (38). They also re-analyzed Karim *et al.*’s crystallographic data and proposed a possible model containing a DNA duplex (PDB: 7ABS).

Recently, important cryo-EM work by Gellert and Yang revealed the complex of Artemis:DNA-PKcs:Ku70/80:DNA (PDB: 7SGL) (6). It provides the first look at the Artemis catalytic domain binding to a DNA end in context of the DNA-PKcs:Ku70/80:DNA complex and describes important conformational changes in Artemis that are required for DNA end processing during NHEJ. The catalytic domain in this structure is located within the HEAT cradle bound to the DNA end and contains two Mg^2+^ instead of Zn^2+^ at the active site. Also revealed in this structure is a short portion of the C-terminal tail of Artemis (E366–P406) bound to the FAT domain of DNA-PKcs. Using the previous finding that Artemis and DNA-PKcs co-immunoprecipitation is DNA-independent (7), Niewolik and Schwarz defined 26 amino acids of Artemis (378–403 aa) as a minimal DNA-PKcs interacting fragment (39).

Since the majority of Artemis appears to exist in complex with DNA-PKcs within cells (7), it has not been clear how Artemis alone exists in cells. Whether it exists as a monomer, a dimer, or multimers, or by forming a complex with other proteins, such as Ligase IV has been uncertain. Here, we use cryo-EM to analyze the basal state of Artemis:DNA-PKcs endonuclease complex. This is the state of the complex after purification, but prior to, activation with ATP and a DNA end and presumably the state that would be present in the cell before the complex is recruited to broken DNA ends. We determine the position of Artemis on DNA-PKcs prior to activation of the kinase activity of DNA-PKcs. The N-terminal catalytic domain of Artemis is dynamic, even in complex with DNA-PKcs in this basal state. The data permit a detailed structural model of the Artemis tail bound on DNA-PKcs with proposed interactions between Artemis and DNA-PKcs, demonstrating why a SCID human mutation, L3062R, of DNA-PKcs may interfere with Artemis interaction. Our study permits structure-guided drug development against the basal state.

## MATERIALS AND METHODS

### Purification of endogenous DNA-PKcs

Endogenous native human DNA-PKcs was purified to near homogeneity from HeLa-S3 cells as follows. The frozen cell pellet was produced by the Cell Culture Company, LLC (Minneapolis, MN), using our specifications for cell growth rate and density. The nuclear extract was prepared using a modified protocol based on the method of Dignam (40). Chromatography resins included DEAE Sepharose FF, Heparin Sepharose 6 FF, dsDNA(oligo) CNBr-activated Sepharose 4 FF, Mono Q 5/50 GL, Mono S 5/50 GL and Superdex 200 Increase 10/300 GL, purchased from Cytiva (Marlborough, MA). Unless otherwise indicated, all buffer solutions used for chromatography were filtered with 0.2 μm filters and degassed. For dialysis of protein samples, a Biotech CE Dialysis Tubing 100 kD 3.1 ml/cm membrane (Spectrum Laboratories, Inc., Rancho Dominguez, CA) was used. Additional protease inhibitors mentioned below specifically indicate a mixture of 2 μg/ml aprotinin, 1 μg/ml pepstatin A, 10 μM leupeptin, 1 μM bestatin and 10 μM E-64 which were purchased from Sigma-Aldrich Corp. (St. Louis, MO).

The frozen cells were thawed in a 37°C water bath while keeping the cells cold. The rest of the procedures were performed at 4°C or on wet ice. The thawed cells were rapidly resuspended in 5× packed cell volume of NE Buffer A-K10: 10 mM HEPES–KOH (pH 7.9), 10 mM KCl, 1.5 mM MgCl_2_, 0.2 mM PMSF, 5 mM DTT and additional protease inhibitors. The resuspended cells were centrifuged at 1,850 ×g for 5 min and the pellets were kept. The supernatant was further centrifuged at 10,000 ×g for 10 min. All the cell pellets were resuspended in NE Buffer A-K10 supplemented with additional protease inhibitors to a final volume of 3× the original packed cell volume. The resuspended cells were incubated on ice for 10 min to swell the cells. The cells were homogenized with a glass Dounce homogenizer (a type B pestle) with 15 strokes. The efficiency and completion of cell lysis (>∼90%) were examined under a microscope by trypan blue staining. The homogenized cells were centrifuged at 3,200 ×g for 15 min and the pellets (i.e. nuclei) were kept. The supernatant was further centrifuged at 25,000 ×g for 20 min and the pellets (i.e. nuclei) were collected. All the nuclei pellets were resuspended in a volume of NE Buffer B-Na20: 10 mM HEPES–NaOH (pH 7.9), 20 mM NaCl, 1.5 mM MgCl_2_, 0.2 mM EDTA, 25% (v/v) glycerol, 0.2 mM PMSF, 5 mM DTT and additional protease inhibitors, equal to half of the packed nuclear volume. In a dropwise fashion, while stirring gently, an appropriate volume of NE Buffer C-Na840: 20 mM HEPES–NaOH (pH 7.9), 840 mM NaCl, 1.5 mM MgCl_2_, 0.2 mM EDTA, 25% (v/v) glycerol, 0.2 mM PMSF, 5 mM DTT and additional protease inhibitors, was added to the sample to reach ∼420 mM NaCl as the final concentration. The resuspended nuclei were homogenized with the Dounce homogenizer (a type B pestle) with five strokes. The homogenizer was rinsed with NE Buffer D-Na420: 20 mM HEPES-NaOH (pH 7.9), 420 mM NaCl, 1.5 mM MgCl_2_, 0.2 mM EDTA, 25% (v/v) glycerol, 0.2 mM PMSF, 5 mM DTT and additional protease inhibitors, to recover as much sample as possible. The nuclei suspension was gently and continuously mixed on an end-over-end rotor for 40 min. The mixed sample was centrifuged at 25,000 ×g for 30 min. At this point, the supernatant (i.e. high salt nuclear extract) was removed and dialysis was started against DEAE-80 Dialysis Buffer: 50 mM Tris–HCl (pH 7.5), 80 mM KCl, 0.5 mM EDTA, 5% (v/v) glycerol, 0.2 mM PMSF and 5 mM DTT. The nuclear pellets were gently resuspended or rinsed with NE Buffer D-Na420 and mixed on an end-over-end rotor for 30 min. The mixed sample was centrifuged at 25,000 ×g for 30 min. The supernatant (i.e. high salt nuclear extract) was dialyzed against DEAE-80 Dialysis Buffer: 50 mM Tris–HCl (pH 7.5), 80 mM KCl, 5 mM EDTA, 5% (v/v) glycerol, 0.2 mM PMSF and 5 mM DTT. The dialyzed nuclear extract was centrifuged at 25,000 ×g for 30 min. The supernatant was supplemented with additional protease inhibitors.

This nuclear extract was passed over the DEAE Sepharose FF column (XK50/20: CV = ∼250 ml), which was equilibrated with DEAE-90 Buffer: 50 mM Tris–HCl (pH 7.5), 90 mM KCl, 0.5 mM EDTA, 5% (v/v) glycerol, 0.2 mM PMSF and 5 mM DTT, using a peristaltic pump. DNA-PKcs was eluted with DEAE Buffer with ∼270 mM KCl. The pooled sample was dialyzed against Heparin-35 Dialysis Buffer: 50 mM Tris–HCl (pH 7.3), 35 mM KCl, 0.5 mM EDTA, 5% (v/v) glycerol, 0.2 mM PMSF and 5 mM DTT, down to ∼113 mM KCl. The dialyzed sample was supplemented with additional protease inhibitors. The post-DEAE sample was passed over the Heparin Sepharose 6 FF column (XK50/20: CV = ∼200 ml), which was equilibrated with Heparin-116.5 Buffer: 50 mM Tris–HCl (pH 7.3), 116.5 mM KCl, 0.5 mM EDTA, 5% (v/v) glycerol, 0.2 mM PMSF and 5 mM DTT. DNA-PKcs was eluted with Heparin Buffer with ∼668 mM KCl. The pooled sample was dialyzed against dsDNA-35 Dialysis Buffer, which is equivalent to the Heparin-35 Dialysis Buffer. The dialyzed sample was supplemented with additional protease inhibitors. The post-Heparin sample was passed over the dsDNA(oligo) Sepharose 4 FF column (XK26/20: CV = ∼40 ml), prepared with a duplex DNA (GW1/2) (see Supplementary Table S1 for the sequence), which was equilibrated with dsDNA-50 Buffer: 50 mM Tris–HCl (pH 7.3), 50 mM KCl, 0.5 mM EDTA, 5% (v/v) glycerol, 0.2 mM PMSF and 5 mM DTT. DNA-PKcs was eluted with dsDNA Buffer with ∼411 mM KCl. The pooled sample was dialyzed against QS-35 Dialysis Buffer: 50 mM Tris–HCl (pH 7.5), 35 mM KCl, 0.5 mM EDTA, 5% (v/v) glycerol, 0.02% (v/v) Tween-20, 0.2 mM PMSF and 5 mM DTT. The dialyzed sample was centrifuged at 25,000 ×g for 20 min and filtered with a 0.2 μm Supor Membrane Acrodisc Syringe Filter (25 mm disc) (Pall Corporation, Port Washington, NY). The filtered sample was injected into the Mono Q 5/50 GL column, which was equilibrated with QS-100 Buffer: 50 mM Tris–HCl (pH 7.5), 100 mM KCl, 0.5 mM EDTA, 5% (v/v) glycerol, 0.02% (v/v) Tween-20, 0.2 mM PMSF and 5 mM DTT. After washing, the column was subjected to linear gradients with QS-1M Buffer: 50 mM Tris–HCl (pH 7.5), 1 M KCl, 0.5 mM EDTA, 5% (v/v) glycerol, 0.02% (v/v) Tween-20, 0.2 mM PMSF and 5 mM DTT. In the region of a 10–20% QS-1 M Buffer linear gradient, two samples were separately pooled: one is the fractions that contained the majority of DNA-PKcs and the other is the fractions that contained some portion of DNA-PKcs and Ku70/80. Each pooled sample was applied onto Econo-Pac^®^ 10 DG columns (Bio-Rad Laboratories, Inc., Hercules, CA), which were equilibrated with QS-100 Buffer, to desalt. The desalted sample which contained the majority of DNA-PKcs was further purified with the Mono S column. The other desalted sample which contained some portion of DNA-PKcs and Ku70/80 was saved to purify native Ku70/80 later. The sample which contained the majority of DNA-PKcs was injected into the Mono S 5/50 GL column, which was equilibrated with QS-100 Buffer. After washing, DNA-PKcs was eluted in a 0–15% QS-1M Buffer linear gradient. The pooled sample was concentrated down to ∼500 μl using Vivaspin 500 (10 K MWCO) concentrators (Satorius Stedim Biotech GmbH, Goettingen, Germany) and the centrifuged at 16,000 rcf for 10 min. The centrifuged sample was injected into the Superdex 200 Increase 10/300 GL column, which was equilibrated with S200 Buffer11: 25 mM Tris–HCl (pH 7.5), 200 mM NaCl and 10 mM DTT, at a flow rate of 0.75 ml/min.

For cryo-EM purposes, a small amount of the DNA-PKcs peak fraction was diluted with 25 mM Tris–HCl (pH 7.5), 100 mM NaCl and 2 mM DTT to adjust the concentration to ∼1 μM based on the *A*_280_ value-based estimation. The final sample was freshly used for plunge-freezing and some were snap frozen in liquid nitrogen and stored at –80°C.

The rest of the peak fractions were diluted with the equal volume of 25 mM Tris–HCl (pH 7.5) and concentrated using Vivaspin 500 (10 K MWCO) concentrators (Satorius Stedim Biotech GmbH, Goettingen, Germany). The concentrated sample was centrifuged at 16,000 rcf for 10 min. The protein concentration was determined by the method of Bradford using Bio-Rad Protein Assay dye reagent (Bio-Rad Laboratories, Inc., Hercules, CA) with bovine serum albumin as a standard. The final concentration was adjusted appropriately, and small aliquots were snap frozen in liquid nitrogen and stored at –80°C. This whole procedure from thawing the cell pellet, harvested from 16 L of cell culture, to storing the final protein aliquots was done over 3 days.

### Purification of endogenous Ku70/80

The saved MonoQ fractions, which contained some portion of DNA-PKcs and Ku70/80 above, were injected onto the Mono S 5/50 GL column, which was equilibrated with QS-100 Buffer and the flowthrough, which contained the majority of Ku70/80, was collected. The buffer of the flowthrough was exchanged using Econo-Pac^®^ 10 DG columns (Bio-Rad Laboratories, Inc., Hercules, CA), with QS-100 Buffer#2: 50 mM Tris–HCl (pH 7.5), 100 mM KCl, 0.5 mM EDTA, 5% (v/v) glycerol, 0.2 mM PMSF and 5 mM DTT, to remove Tween-20. The sample was injected onto the Mono S 5/50 GL column, which was equilibrated with QS-100 Buffer#2. After washing, the column was subjected to linear gradients with QS-1M Buffer#2: 50 mM Tris–HCl (pH 7.5), 1 M KCl, 0.5 mM EDTA, 5% (v/v) glycerol, 0.2 mM PMSF and 5 mM DTT. In the region of a 10–20% QS-1 M Buffer#2 linear gradient, Ku70/80 was eluted. The peak fractions were concentrated down to ∼500 μl using Vivaspin 20 and Vivaspin 500 (both 10 K MWCO) concentrators (Satorius Stedim Biotech GmbH, Goettingen, Germany) and the concentrated sample was centrifuged at 16,000 rcf for 10 min. The centrifuged sample was injected into the Superdex 200 Increase 10/300 GL column, which was equilibrated with S200 Buffer11: 25 mM Tris–HCl (pH 7.5), 200 mM NaCl and 10 mM DTT, at a flow rate of 0.75 ml/min. The peak fractions were concentrated using Vivaspin 500 (10 K MWCO) concentrators (Satorius Stedim Biotech GmbH, Goettingen, Germany) and the concentrated sample was centrifuged at 16,000 rcf for 10 min. Once the concentration was determined by Bradford assay, Ku70/80 was aliquoted into a small volume, flash frozen in liquid nitrogen and stored at –80°C.

### Production of recombinant Artemis

The codon of the human Artemis coding sequence was optimized for *Trichoplusia ni* and the synthesized gene, with the 3′ flanking sequence for a TEV cleavage site and (8×)His tag, was cloned into pFastBac1 at the BamHI and HindIII sites (GenScript USA Inc., Piscataway, NJ). The plasmid containing Artemis cDNA + TEV+(8x)His was transformed into DH10Bac competent cells (Thermo Fisher Scientific Inc., Waltham, MA), and the generated bacmid was isolated. The baculovirus was generated by transfecting the generated bacmid into Sf21 cells. The isolated baculovirus was amplified in Sf21 cells and the expression of Artemis was confirmed in both Sf21 and High Five™ cells (Thermo Fisher Scientific Inc., Waltham, MA). The titrated virus was used to infect High Five cells for protein production in a large scale. The infected cells were cultured shaking at ∼130 rpm at 27°C and harvested at 48 h of post-infection, flash-frozen in liquid nitrogen and stored at –80°C.

### Purification of recombinant Artemis

The frozen High Five cell pellet harboring recombinant human Artemis-(8×)His was thawed at 37°C, while keeping the cells cold. The rest of the procedures were performed at 4°C. The thawed cells were resuspended in TALON Binding Buffer: 50 mM HEPES–NaOH (pH 7.9), 500 mM NaCl, 5% (v/v) glycerol, 10 mM imidazole, 0.1% (v/v) Triton X-100, 1 mM TCEP, 0.2 mM PMSF, 2 μg/ml aprotinin, 1 μg/ml pepstatin A, 10 μM leupeptin, 1 μM bestatin and 10 μM E-64. The cells were sonicated and centrifuged at 25,000 ×g for 30 min. The supernatant was incubated with TALON^®^ Superflow Metal Affinity Resin (Takara Bio USA, Inc., San Jose, CA) resin, pre-washed with TALON Binding Buffer, and mixed by rotating end over end for 6 h. After incubation, the resin was washed with TALON Binding Buffer with 800 mM NaCl twice. The washed resin was transferred into an empty Econo-Pac^®^ gravity-flow column (Bio-Rad Laboratories, Inc., Hercules, CA), and further washed with TALON Wash Buffer: 50 mM HEPES–NaOH (pH 7.9), 800 mM NaCl, 5% (v/v) glycerol, 50 mM imidazole, 0.005% (v/v) Triton X-100, 1 mM TCEP, 0.2 mM PMSF, 2 μg/ml aprotinin, 1 μg/ml pepstatin A, 10 μM leupeptin, 1 μM bestatin and 10 μM E-64. Artemis was eluted with TALON Elution Buffer: 50 mM HEPES–NaOH (pH 7.9), 500 mM NaCl, 5% (v/v) glycerol, 250 mM imidazole, 0.005% (v/v) Triton X-100, 1 mM TCEP, 0.2 mM PMSF, 2 μg/ml aprotinin, 1 μg/ml pepstatin A, 10 μM leupeptin, 1 μM bestatin and 10 μM E-64. The eluted Artemis was desalted with Econo-Pac^®^ 10DG columns (Bio-Rad Laboratories, Inc., Hercules, CA), which were equilibrated with S200 Buffer: 25 mM Tris–HCl (pH 7.5), 200 mM NaCl, 2 mM DTT and 0.005% Triton X-100. The desalted sample was concentrated down to ∼500 μl using Vivaspin 20 and Vivaspin 500 (both 10 K MWCO) concentrators (Satorius Stedim Biotech GmbH, Goettingen, Germany) and the concentrated sample was centrifuged at 16,000 rcf for 10 min. The centrifuged sample was injected into the Superdex 200 Increase 10/300 GL column, which was equilibrated with S200 Buffer without Triton X-100 at a flow rate of 0.75 ml/min. Depending on the height of the peak, the peak fraction was run onto the second round of size exclusion column.

For cryo-EM purposes, a small amount of the Artemis peak fraction was freshly used for complex formation with DNA-PKcs and plunge-freezing. The rest of the peak fractions were supplemented with 0.005% (v/v) Triton X-100, flash frozen in liquid nitrogen and stored at –80°C.

The purity of purified proteins was assessed by Coomassie Brilliant Blue R250 staining or silver staining on 6 or 8% SDS-PAGE using a Spectra™ Multicolor High Range Protein Ladder (Thermo Fisher Scientific Inc., Waltham, MA) or BLUEstain^TM^ Protein Ladder (Gold Biotechnology, St Louis, MO) as a marker as indicated.

### Preparation of a Y-shape duplex DNA (GW132H)

An oligo for Y-shape double hairpins DNA, GW132 (see Supplementary Table S1 for the sequence), received from Integrated DNA technologies, Inc. (Coralville, IA), was first purified by 5% denaturing PAGE with 7 M urea at room temperature. The DNA extracted from gel was purified further through Mono Q 5/50 GL (Cytiva, Marlborough, MA) using 10 mM NaOH with NaCl gradient. The eluted DNA was neutralized with 1 M HEPES–NaOH (pH 7.5), dialyzed against ddH_2_O and lyophilized. The lyophilized oligo was dissolved in dsDNA Annealing Buffer: 20 mM HEPES–NaOH (pH7.5), 100 mM NaCl and 10 mM MgCl_2_ and self-annealed. Finally, the quality of dsDNA, as a Y-shape form (GW132H), was assessed by running 20% native PAGE at 4°C. GW132H is configured to be blocked with two short hairpins at one end (creating a Y-shape at this end) and two short overhangs (1 and 3 nt 3′ and 5′ overhangs, respectively) at the open DNA end.

### Agarose-acrylamide composite gel shift assay

An agarose-acrylamide composite native gel shift assay system was further optimized from a method of Suh *et al.* (41). The gel system used was Mini-PROTEAN^®^ II Electrophoresis Cell (Bio-Rad Laboratories, Inc., Hercules, CA) with a 1.5 mm space thickness. This procedure describes the making of two gels. First, for each apparatus, ∼2 ml of 20% polyacrylamide gel was prepared from a 30% acrylamide/bis-acrylamide (29:1 (w/w)) stock solution (30%T/3.33%C) and 10× TBE stock solution on the bottom of the gel. About 25 μl of 10% (w/v) ammonium persulfate and 4.5 μl of TEMED were added to make ∼5 ml of 20% polyacrylamide solution. The 2 ml of acrylamide solution was equivalent to ∼1.4 cm of height of the gel from the bottom. For the agarose, SeaKem^®^ Gold Agarose was used (Lonza Rockland, Inc., Rockland, ME). Then, a solution of 1.0% (w/v) agarose (e.g. 0.15 g in 15 ml of 1× TBE) was made in a 125-mL Erlenmeyer flask by heating in a microwave (1,000 W) for 1 min and swirling every 20 s to promote mixing and melting of the agarose. The flask was loosely sealed with aluminum foil and kept in a 45°C water bath to prevent gelation. Then, the acrylamide solution was prepared in another 125-ml Erlenmeyer flask by adding and mixing the following in this order: 8.16 ml of ddH_2_O, 2.0 ml of 30% acrylamide/bis-acrylamide (75:1 (w/w)) stock solution (30%T/1.32%C), 3.0 ml of 10× TBE, 1.5 ml of 50% (w/v) glycerol, 15 μl of 1 M DTT and 300 μl of 10% (w/v) ammonium persulfate. The mixed solution was degassed for 1 min while swirling, loosely sealed with aluminum foil, and then transferred to the same 45°C water bath for 20 min to reach thermal equilibration. The gel apparatus, which contained the 20% polyacrylamide gel, was half filled with diH_2_O and placed in a 37°C incubator to pre-warm the glass plates until the 20 min incubation of the acrylamide solution in the water bath ends. After 20 min incubation, the warmed acrylamide solution was poured into the flask containing the molten agarose, along with condensed water on the inside wall of this flask and mixed by gentle swirling. The mixture was quickly poured back to the other flask, which had contained the acrylamide solution, along with condensed water on the inside wall of this flask, mixed by gentle swirling and incubated at 45°C for 1 min. Then, after adding 30 μl of TEMED, the gel mix was rapidly but gently poured into the pre-warmed gel assembly (×2), a short 10-well comb was carefully inserted without air bubbles, and the assembly was immediately transferred to 4°C for 10 min for the agarose to solidify. The cooled gel assembly was taken out after 10 min and kept at room temperature for 1 h to allow full polymerization of the acrylamide. This agarose gelation step prior to polymerization is essential, providing a vital mechanical support to the composite gel since the polymerized acrylamide alone would still be fluid-like at this low concentration (e.g. 2%). Then, after 1 h incubation at room temperature, the comb was gently removed with extreme care. The shape of wells was polished with a spatula if necessary. Creating wells with a very flat base was extremely important because of the nature of native gel electrophoresis. Also trimming of the wells must be done carefully because of the fragility of the composite gel and its glutinous nature. But the polishing step must be completed before the gel starts to dry. After the polishing, the gel apparatus was assembled with cold 1× TBE as a running buffer. No air bubbles at the bottom of the gel should be trapped. Usually, the apparatus was kept at 4°C overnight to pre-cool. Then, gel shift assays were carried out at 4°C.

The binding reaction was carried out in a final volume of 10 μl with a buffer containing 20 mM Tris–HCl (pH 7.5), 100 mM NaCl, 2 mM DTT, 5% (v/v) glycerol, 0.3 μM DNA-PKcs, 0.3 μM Ku70/80, 0.3 μM Artemis, 0.36 μM Y-shape duplex DNA GW132H as indicated at 4°C for 30 min. The assembly of each complex was done by incubating them at 4°C for another 30 min. For example, for the assembly of DNA-PK, 0.3 μM DNA-PKcs was added to the complex of Ku70/80:GW132H and incubated at 4°C for another 30 min. Artemis was added to each appropriate complex and incubated at 4°C for another 30 min. During this incubation, the gel was pre-run at 100 V for 30 min. After the complex assembly reactions, the samples were run onto an agarose-acrylamide composite native gel at 4°C at 100 V for 2 h. After the run, the gel was stained with silver nitrate. To visualize free DNA, the gel was overdeveloped.

### Peptide competition assay

An Artemis peptide (Art: LRHKVPY) and an XRCC4 peptide (X4: APSRKRRQRMQR) were synthesized and analyzed by GenScript USA Inc. (Piscataway, NJ). The N-terminus and C-terminus of both peptides were acetylated and amidated, respectively. Various concentrations of peptides (e.g. 0 μM, 30 μM, 300 μM and 3000 μM) were added into pre-formed Artemis:DNA-PKcs complex, as described above, and incubated at 4°C for another 30 min. Also, to assess whether Artemis binds DNA-PKcs to form a complex in the presence of the XRCC4 peptide, 0.3 μM DNA-PKcs and the 3 mM XRCC4 peptide were mixed and incubated at 4°C for 30 min first, and then, 0.3 μM Artemis was added and incubated at 4°C for another 30 min prior to the gel run. Controls included individual peptides and proteins and the combination of them. The samples were run on an agarose-acrylamide composite native gel at 4°C at 100 V for 2 h. After the run, the gel was stained with silver nitrate.

### Artemis nuclease assay

The sequence of the oligos is listed in Supplementary Table S1. The Artemis nuclease assay was performed as previously described (42), using the Artemis substrate, ZE16, which is fluorescently labeled at the 5′ end and self-anneals to form an 8-nt 5′ overhang, with the first four nucleotides with phosphorothioate bonds to prevent degradation by the exonuclease activity of Artemis, and a 12-bp double-stranded hairpin portion. For the Mn^2+^ assay, 10 nM Artemis without DNA-PKcs was used to initiate the reaction in the buffer contained 25 mM Tris–HCl (pH 8.0), 10 mM KCl, 10 mM MnCl_2_, 500 nM ZE16 and 1 mM DTT. For the Artemis:DNA-PKcs with ATP/Mg^2+^ assay, an equal molar ratio of Artemis and DNA-PKcs was pre-incubated together on ice for 10 min to form a complex. Then, the reaction, which contained 25 mM Tris–HCl (pH 8.0), 10 mM KCl, 100 μM ATP, 10 mM MgCl_2_, 500 nM ZE16 and 1 mM DTT, was initiated by adding 10 nM Artemis:DNA-PKcs complex and incubating at 37°C for 30 min. The reactions were stopped by the addition of an equal volume of 1:1 40% glycerol:formamide and incubation at 95°C for 5 min. Reaction products were resolved with an 18% denaturing PAGE. A 32 nt marker oligonucleotide (GW35) and an 8 nt marker oligonucleotide (FAM-8 T) were used to aid gel mobility determination.

### Electron-microscopy sample preparation and screening

Cryo-grid preparation was performed using a home-made manual plunger at a typical indoor humidity of 73% and at room temperature. Briefly, UltrAuFoil R 1.2/1.3 grids (Gold 300 mesh) (Quantifoil Micro Tools GmbH, Jena, Germany) were glow-discharged using a PELCO easiGlow™ Discharge System (Ted Pella, Inc., Redding, CA) for 40 s on both sides at 0.3 mBar and 20 mA. The Artemis:DNA-PKcs complex was prepared by mixing them at 1.12× molar excess Artemis to a final complex concentration of ∼280 nM and incubating them at 4°C for 30 min. The Artemis:DNA-PKcs:GW132H complex was prepared by incubating DNA-PKcs and 1.12× molar excess GW132H at 4°C for 30 min first, and subsequently adding 1.12× molar excess Artemis to DNA-PKcs to a final complex concentration of ∼280 nM and incubating at 4°C for another 30 min. Then, LMNG was supplemented to both samples at the final concentration of 5.5 nM. The final samples were evenly applied onto both sides of the glow-discharged grids (1 μl and 2 μl). A piece of filter paper (Grade 41 Fast Ashless Whatman Filter Paper) (Cytiva, Marlborough, MA) was applied to one side of the grid to adsorb the sample for ∼3 s. Then, the grid was quickly plunged into liquid ethane, pre-cooled with liquid nitrogen. The plunged-grids were taken to UCI Irvine Materials Research Institute and screened with a JEOL JEM-2100F TEM at 200 kV, equipped with Gatan OneView and K3 cameras. The optimal grid candidates were shipped to the National Cryo-Electron Microscopy Facility (NCEF) of the National Cancer Institute (NCI) for data collection.

### Image acquisition and processing for Artemis:DNA-PKcs and Artemis:DNA-PKcs:DNA

At NCEF, movies of the Artemis:DNA-PKcs complex were collected using a Titan Krios operating at 300 kV equipped with a Gatan K3 camera behind a bioquantum imaging filter with an energy filter slit width of 20 eV. For Artemis:DNA-PKcs data, four datasets were collected: one dataset with the super-resolution mode at 0.54 Å/pixel and a defocus range of −1.0 to −2.5 μm and 3 datasets with the counting mode at 1.08 Å/pixel and a defocus range of −0.75 to −3.0 μm. The magnification was 81,000× for all datasets and for each movie, a total exposure dose of 60 e^–^/Å^2^ was fractionated into 40 frames for 3.25 s.

For Artemis:DNA-PKcs:GW132H complex, two datasets were collected with the counting mode at 1.08 Å/pixel and a defocus range of −0.75 to −2.25 μm. The magnification was 81,000× and for each movie, a total exposure dose of 45 e^–^/Å^2^ was fractionated into 40 frames for 3.25 s.

Suboptimal movie files were excluded based on Scipion output first and the remaining movies were imported into cryoSPARC (v3.2.0 + 210511) (43). Patch motion correction was performed, excluding the first 2 frames and the last 10 frames of each image stack. For the dataset with the super-resolution mode, a Fourier cropping factor of $\frac{1}{2}$ was applied. After Patch CTF estimation, images were curated based on CTF fit resolution <10.0, relative ice thickness <1.50 and total full-frame motion distance <30.0. Then, a blob picker job was used to pick particles. A total 2,819,901 particles were extracted from 15,659 images with a box size of 512 × 512 pixels. Extraneous materials including contaminants and overlapped particles were removed from rounds of 2D classification. A combination of multi-class Ab-initio reconstruction, heterogeneous refinement and additional rounds of 2D classification was used to get rid of irrelevant particles including DNA-PKcs alone particles. Then, 339,945 particles were subjected to a single-class Ab-initio reconstruction. This initial map was refined with homogeneous refinement to 3.16 Å, followed by a local refinement with non-uniform refinement to obtain 3.09 Å (FSC at 0.143 cutoff) (44). Further rounds of heterogeneous refinements were performed to obtain three different maps (or states) with acceptable particles numbers. For each state, additional rounds of 2D classification were performed and improved maps were obtained from homogeneous refinement. Local CTF refinement and global CTF refinement were performed to improve resolution (45). Then a mask was created using Chimera for each of three maps and local refinement was performed with the corresponding mask, resulting in 3.48 Å for the state 1, 3.33 Å for the state 2 and 3.42 Å for the state 3 (FSC at 0.143 cutoff). For each map, sharpening tools were used with various *B*-factors to obtain reasonable maps including blurred maps. Also, 3D variability analysis was performed (46). The blurred map (sharpened a *B*-factor at +80 Å^2^) of the state 2 was used to assist locating the Artemis catalytic part. The head part of DNA-PKcs (FAT + kinase domains) with Artemis tail were segmented out using Segger in Chimera (47) and masks on each were created for further local refinement. Although the resolution was improved, the reliability and interpretation of map quality were not improved.

### Model building and refinement for Artemis:DNA-PKcs complex

Atomic coordinates of a crystal structure of DNA-PKcs (PDB: 5LUQ) were fit into preliminary maps, from intermediate steps of image processing (28). Amino acid registrations were manually fixed to create a preliminary model in *Coot* (48). Then, once the final map was obtained (e.g. State 2 map), the preliminary model was fit into the map for further refinement. The overall model was compared with more recent cryo-EM structures (PDB: 6ZFP) later on to assess any disagreement (33). A density modification was applied to improve the map using ResolveCryoEM in Phenix (dev-4383) (49–51). The resolution was calculated to 3.18 A (FSC at 0.5 cutoff). Using both maps of the state 2, the density modified map and blurred map from the cryoSPARC, the model was built. After rounds of real space refinement in Phenix with iterative investigation and manual assignment of secondary structure restraints for DNA-PKcs, the final model which contains DNA-PKcs and part of the Artemis tail was created. Molprobity in Phenix, wwPDB Validation Service (https://validate.wwpdb.org) and the Quality Control Check (https://qc-check.usc.edu) were used to validate the model quality. The refinement statistics were shown in Supplementary Table S2.

The crystal structure of the Artemis catalytic domain (PDB: 7AF1) was manually fit into a globular density external to DNA-PKcs of the blurred map. The best orientation was chosen from four possible orientations of the catalytic domain. Flex-EM in CCP-EM v1.5.0 was performed to assess the fit quality for four possibilities for vertical reversal for the catalytic domain of Artemis (52,53).

The contacts and distances between atoms are calculated with CONTACTS in CCP4 v7.1.018 (54). RMSD was calculated with PyMOL (The PyMOL Molecular Graphics System, Version 2.0 Schrödinger, LLC.). The figures were created using PyMOL, Chimera (55) and ChimeraX (56,57).

### CryoDRGN analysis

Variation autoencoder methodology incorporated in cryoDRGN was employed to assess structural variability in the data and to provide a greater understanding of the continuous motions of Artemis relative to DNA-PKcs (58). A consensus reconstruction using 251,011 DNA-PKcs, Artemis particles was one of three heterogeneous reconstructions that had the strongest Artemis density and a GSFSC resolution of 3.2 Å resolution. Poses and CTF parameters were derived from a subsequent homogeneous refinement of this subset that attained 3.9 Å^2^ unmasked GSFSC in cryoSPARC. The decoder network was trained on this dataset with 1.08 Å^2^ pixel, a 256^2^ pixel dimension, a z-dimension of 8, encoder space of 1024 × 3, over 25 epochs. Encoder output volumes presented from this work were selected from Umap latent space or PCA traversals as specified.

### RoseTTAFold analysis

A full-length Artemis amino acid sequence was used for RoseTTAFold analysis to predict an overall structure of Artemis including the C-terminal tail (59).

### AlphaFold analysis

A predicted Artemis structure by AlphaFold v2.0 was obtained from the AlphaFold Protein Structure Database (60).

## RESULTS AND DISCUSSION

### Gel and cryo-EM visualization of Artemis:DNA-PKcs complex

**Figure 1.**
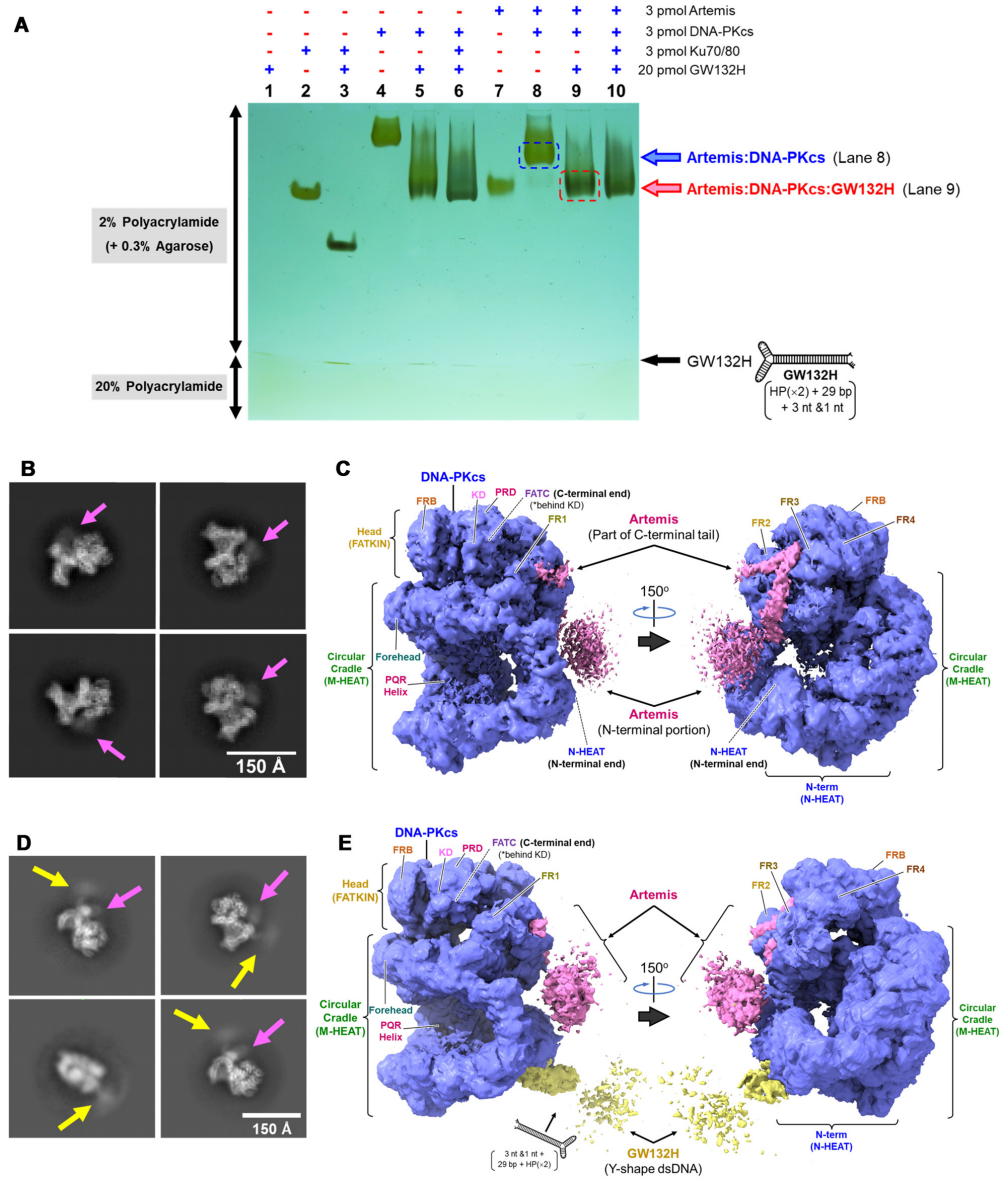
Gel and cryo-EM classification of the Artemis:DNA-PKcs complex. (**A**) Agarose-acrylamide composite gel shift assay. The binding reaction was carried out in a final volume of 10 μl with a buffer containing 20 mM Tris–HCl (pH 7.5), 100 mM NaCl, 2 mM DTT, 5% (v/v) glycerol with 0.3 μM DNA-PKcs, 0.3 μM Ku70/80, 0.3 μM Artemis and 0.36 μM Y-shape duplex DNA GW132H as indicated at 4°C for 30 min. The complexes of Artemis:DNA-PKcs and Artemis:DNA-PKcs:DNA are highlighted (lanes 8 and 9, respectively). For the assembly of DNA-PK (lanes 6 and 10), 0.3 μM DNA-PKcs was added to the complex of Ku70/80:GW132H and incubated at 4°C for another 30 min. Artemis was added to each complex (lanes 9 and 10) and incubated at 4°C for another 30 min. The samples were run on an agarose-acrylamide composite native gel, composed of 2.0% polyacrylamide and 0.3% agarose, at 4°C. The gel was stained with silver nitrate and overdeveloped to visualize free DNA trapped in a 20% polyacrylamide gel in the bottom portion. A visual description of the Y-shape form of GW132H is shown on the right side of the gel. (**B**) 2D Classification of Artemis:DNA-PKcs complex. Representative Artemis:DNA-PKcs class averages generated in cryoSPARC are shown. Scale bar, 150 Å. Magenta arrows point at an extra density to DNA-PKcs, which indicates the presence of Artemis in some classes. (**C**) Overall cryo-EM map of Artemis:DNA-PKcs complex. A blurred map generated by sharpening at a *B*-factor of +80 Å^2^ by cryoSPARC is shown (under EMD-26192, ‘blurred map’). The contour level was set at 0.12 to encompass the Artemis N-terminal catalytic domain. The DNA-PKcs density is colored in blue and the Artemis density is colored in magenta. (**D**) 2D Classification of Artemis:DNA-PKcs:DNA complex. Representative Artemis:DNA-PKcs:DNA complex averages generated in cryoSPARC are shown. Scale bar, 150 Å. A magenta arrow points at the Artemis density. A yellow arrow points at the Y-shape duplex DNA GW132H. (**E**) Position of Artemis relative to DNA-PKcs:DNA complex. A blurred map generated by sharpening at *B*-factor of +80 Å^2^ by cryoSPARC is shown (under EMD-26198, ‘blurred map’). The contour level was set at 0.065 to encompass the Artemis N-terminal catalytic domain as well as the Y-shape dsDNA (GW132H). The Artemis density is colored in magenta. The Y-shape dsDNA (GW132H) is colored in yellow. The positions of selected domains of DNA-PKcs are labeled in panels (C) and (E) as designated further in Figure 2. Readers are referred to the online version of Figure 1B and D for clearer viewing of the Artemis density in the 2D class images.

Endogenous DNA-PKcs and Ku70/80 purified from HeLa cells and recombinant human Artemis produced from High Five cells (Supplementary Figure S1) were visualized alone and in various complexes on an agarose-acrylamide composite native gel shift assay system (Figure 1A). DNA-PKcs, Ku70/80 and Artemis alone resolved as distinct bands (Figure 1A, lanes 2, 4 and 7). A duplex DNA oligo (GW132H forming Y-shape double hairpins at the closed end and an open pseudo-Y-shape with short overhangs at the open end) (Supplementary Table S1) was used to also identify the position of the complex of Ku70/80:DNA and that of DNA-PKcs:DNA. The configuration of GW132H was designed so that only one molecule of Ku70/80 heterodimer and one of DNA-PKcs can bind on the dsDNA portion (i.e. 29 bp of the stem). The Y-shape was designed so that the Ku70/80 molecule stays bound at the blocked Y-end, instead of allowing Ku70/80 binding at both ends of the DNA. The single stranded portions were added on the double-stranded open end to provide higher kinase activity and stability. The negative charge of the DNA increased the migration rate of the complexes towards the positive electrode at the bottom of the gel (Figure 1A, lanes 3 and 5). The complex of DNA-PKcs:Ku70/80:DNA (Figure 1A, lane 6) migrated slightly faster than the DNA-PKcs:DNA complex (Figure 1A, lane 5). The complex of Artemis:DNA-PKcs migrated faster than DNA-PKcs alone due to the acidic nature of Artemis (Figure 1A, lane 8). The complex of Artemis:DNA-PKcs:DNA migrated slightly slower than that of DNA-PKcs:DNA (Figure 1A, lanes 9 and 5). Moreover, there is no apparent shift of the band of DNA-PKcs:Ku70/80:DNA upon the addition of Artemis (Figure 1A, lanes 6 and 10). Note that the DNA duplex alone (GW132H) can be observed at the interface of the 2% and 20% portions of the polyacrylamide gel (Figure 1A, lanes 3, 5 and 6) and that the duplex DNA became less visible as the DNA at the edge of the gel tends to diffuse out during silver staining steps (Figure 1A, lanes 1 and 10). Ku70/80 and the DNA oligo were only included in Figure 1, and the primary focus of our study is on the ‘basal state,’ which we define for Artemis:DNA-PKcs endonuclease complex as not having seen ATP or DNA ends after purification.

Artemis:DNA-PKcs are believed to reside in a constitutive complex in cells (7,39). Our gel shift assay supports this conclusion as it shows this complex is stable. In order to consider future drug inhibition of Artemis before it becomes activated, it is essential to obtain the structural information of the basal state of the Artemis:DNA-PKcs complex. Our initial crystallographic approaches to obtain high resolution structures were challenging due to the highly dynamic nature of the complexes. Therefore, our stable gel complex of Artemis:DNA-PKcs guided our assembly of a corresponding complex for cryo-EM structure determination. Distinct density attributable to Artemis was found adjacent to recognizable DNA-PKcs molecules after rounds of two-dimensional (2D) classification (Figure 1B). During rounds of extensive 2D classification and heterogeneous refinement, a map with a reasonable resolution of 3.09 Å was obtained (Supplementary Figures S2A and S2B). However, the map still suffered from molecular fluctuation and especially the quality of the N-HEAT of DNA-PKcs was poor. Additional processing steps resulted in three distinct Artemis:DNA-PKcs maps: state 1 at 3.48 Å, state 2 at 3.33 Å, and state 3 at 3.42 Å, at FSC = 0.143 in cryoSPARC. Variation in these three structures appeared mostly attributable to flexibility of the N-HEAT and M-HEAT repeats of DNA-PKcs relative to the FATKIN domain (Supplementary Figures S3A and S3B and Supplementary Videos 1 and 2). In addition, a stronger signal of Artemis catalytic domain was observed in states 1 and 2 (strongest), as described below.

The state 1 complex has the smallest gap of the three complexes between the forehead and M-HEAT (NUC194) and the largest opening between the FAT and N-HEAT domains, ∼60 and ∼34 Å, respectively (Supplementary Figures S3A). In contrast, the state 3 complex has the largest gap between the forehead and M-HEAT (NUC194) and the smallest opening between the FAT and N-HEAT, ∼66 and ∼30 Å, respectively. State 2 is intermediate between states 1 and 3, with ∼64 Å and ∼31 Å for those distances. The backbone and overall RMSD between our state 2 DNA-PKcs structure and Chaplin *et al.*’s state 2 DNA-PKcs (PDB: 6ZFP) is 0.950 Å (11,865 atoms) and 1.095 Å (22,579 atoms), respectively (33). All three states possess density attributable to the flexible C-terminal tail of Artemis (K368–Y407) bound to the FAT domains of DNA-PKcs. Additional but less resolved is the globular density observed in the 2D classes which corresponds to the N-terminal catalytic domain of Artemis, consisting of the MβL and β-CASP domains (approx. 1–360 aa). This density lies externally between the N-terminal HEAT region and the FAT domain of DNA-PKcs.

The state 1 structure contains an additional helical density not observed in states 2 and 3 between the helices (Y2184–G2198 and R2228–C2244) in the M-HEAT domain (Supplementary Figures S3B and S3C). This region has been observed by many groups to have an extra density, but each has assigned different sequences into this density, suggesting this site can accommodate various interacting sequences and is thus an important site of regulatory function or state. This region could be a regulatory hot spot in the DNA-PKcs M-HEAT scaffold and other protein-protein interactions. Specifically, in the DNA-PKcs:DNA structure (PDB: 6ZH8) reported by the Blundell group, this helical density was observed in close proximity to two helices containing residues N2176–G2198 and V2230–D2247. A lack of clear primary structural features led them to assign UNK5009–UNK5016 (UNK refers to unknown or unidentified amino acids) (Supplementary Figure S3C) (33). In the synaptic complex (PDB: 7NFC), the Blundell group assigned to this site a helix (G2721–R2734), which is connected to the YARK-including DNA-binding helix (E2737–Q2765) (34). In a recently published structure of DNA-PKcs:Ku70/80:DNA Complex VII (unphosphorylated, activated) (PDB: 7SU3), reported by Gellert and Yang's group, a fragment of A2720–R2731 was assigned, while in the structure of Artemis:DNA-PKcs:Ku70/80:DNA (PDB: 7SGL), a helix of M1998-A2014 was assigned (6) (Supplementary Figures S4A and S4B). Based on the connectivity and proximity of the DNA-PKcs polypeptide chain, this extra density in our state 1 structure could be a sheet of R1987–P1991, a loop of V1992–P1996, or a helix of M1998–R2012. Among them, the helix (M1998–R2012) seems the most plausible choice due to the reasonable distance. Nevertheless, this site on the M-HEAT domain contributes a broad range of mutually exclusive protein–protein interaction sites, which could be important for a smooth transition between various conformational changes within DNA-PKcs. The role of this site and identification of interacting fragments at various temporal states is interesting and further investigation is required.

Among the three states, the state 2 map had the best resolved tail density and the strongest signal of the catalytic domain and was therefore selected for further refinement and modeling of Artemis bound to DNA-PKcs. To obtain the full expected volume of density of the catalytic domains of Artemis, the map was blurred by sharpening a *B*-factor at +80Å^2^ (Figure 1C). We attribute this to high structural flexibility and a high degree of molecular motion of the Artemis catalytic domain relative to DNA-PKcs even with the subselected particles (Supplementary Figures S2C, S2D and S2F and Supplementary Video 1). Local refinement of the FATKIN domain with the bound Artemis tail produced a map with 2.82 Å at FSC = 0.143; however, the resolution gains did not improve the quality of the Artemis segments bound to the DNA-PKcs FAT domain (not shown).

Gel shift assay of Artemis bound to DNA-PKcs:DNA in the absence of Ku70/80 indicated that a stable complex was also formed (Figure 1A, lane 9). 2D classification revealed the same extra density positioned externally between the N-terminal HEAT region and the FAT domain of DNA-PKcs also when complexed with the GW132H duplex DNA (Figure 1D). Reconstructions from Artemis:DNA-PKcs bound to DNA at 3.77 Å (FSC = 0.143) showed an Artemis density similarly positioned as in the Artemis:DNA-PKcs maps in addition to the density of GW132H DNA (Figure 1E and Supplementary Figures S5A and S5B). Therefore, our results indicate that Artemis remains bound to DNA-PKcs in the presence of a duplex DNA end but does not undergo any significant reorganization with DNA-PKcs in the absence of ATP and therefore, prior to autophosphorylation (Supplementary Figures S5C and S5D and Supplementary Video 3). It is very important to note that, once DNA-PKcs binds dsDNA, this position of the N-terminal catalytic portion of Artemis would prevent Artemis access to the DNA end that is located within the N-HEAT and M-HEAT cradle of DNA-PKcs (6). This would be true also in other DNA-PKcs complexes with Ku70/80:DNA, according to many previous DNA-PKcs:Ku70/80:DNA structures (6,30–34) (and our unpublished work). Importantly, the structure of Artemis:DNA-PKcs:Ku70/80:DNA with the phosphorylated ABCDE showed that the catalytic domain of Artemis does enter inside the HEAT cradle of DNA-PKcs upon the autophosphorylation of the ABCDE cluster and gains access to the DNA end (6).

### Overall structure of the Artemis:DNA-PKcs complex

**Figure 2.**
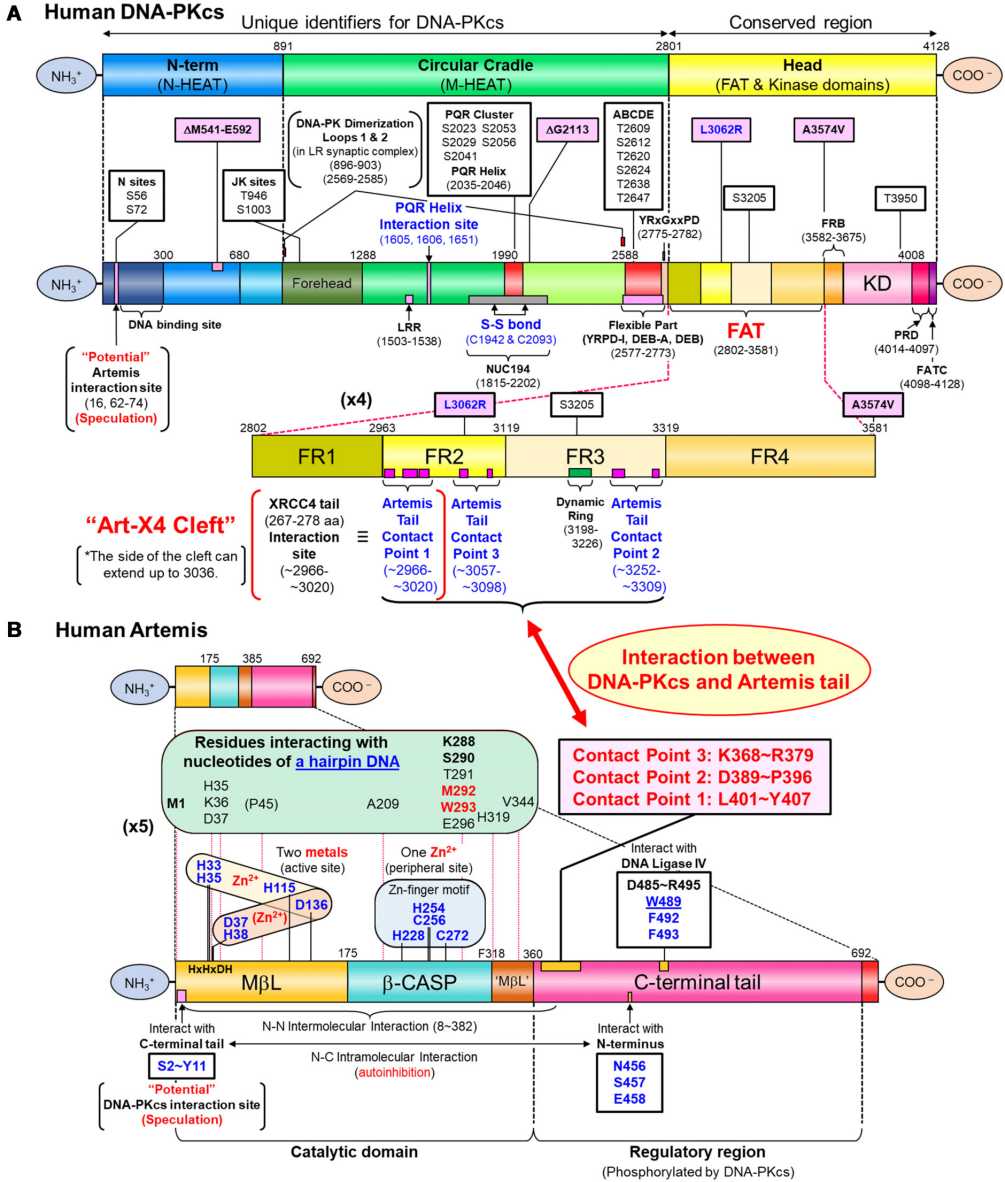
Schematic representation of endogenous human DNA-PKcs and recombinant human Artemis-TEV-(8×)His. (**A**) Schematic representation of endogenous human DNA-PKcs. The top representation shows three segments of DNA-PKcs (4128 amino acids), the N-term (or N-HEAT) region (1–891 aa), the Circular Cradle (or M-HEAT) region (892–2801 aa) and the Head, consisting of FAT and kinase domains (aka FATKIN) (2802–4128 aa). The middle representation shows each segment more in detail. The bottom representation shows ‘4x larger’ FAT domain consisting of the four FR subdomains. The FAT and kinase domains are highly conserved in DNA-PKcs from all Eukaryotic phyla and other PIKKs (TRRAP, SMG1, ATM, ATR and mTOR). On the other hand, the N-HEAT and M-HEAT regions are unique for DNA-PKcs. The N-HEAT region contains dsDNA binding region (stretching R79–K836), accommodating the proximal 15 bp of dsDNA (31). The M-HEAT region contains additional DNA binding sites (e.g. R2311, K2313, K227, R2228) (not shown in figure), the PQR region (2023–2056) and the ABCDE cluster (2609–2647 aa). The region that contains (YRxGxxPD) interacts with the DNA ends. In this study, three Artemis tail contact points are identified in the FAT domain: Contact point 1 (in FR2): ∼2966-∼3018; Contact point 2 (in FR3): ∼3252-∼3309; and Contact point 3 (in FR2): ∼3057-∼3098. The Contact point 1 was also identified as the C-terminal XRCC4 (267–278 aa) interacting site (32) and defined as the Art-X4 cleft. The identified mutations which cause SCID are shown in pink boxes (ΔM541-E592, ΔG2113 + L3062R and A3574V). Select important phosphorylation sites are shown in white boxes (N sites, JK sites, PQR cluster, ABCDE cluster, S3205 and T3950). Newly identified intramolecular interactions are labeled (PQR helix interaction sites and S-S bond sites). The potential Artemis interaction site in the DNA-PKcs N-term is speculative. (**B**) Schematic representation of recombinant human Artemis-TEV-(8×). Human Artemis (692 amino acids) consists of a catalytic domain (1–360 aa), composed of the MβL and β-CASP (CPSF-73-Artemis-SNM1-Pso2) domains, and an unstructured C-terminal regulatory tail (361–692 aa). The catalytic domain forms the two-metal containing active site (two Zn^2+^ ions) at the interface of the MβL and β-CASP domains. One active site Zn^2+^ has strong binding with H33, H35, H115 and D136 and the other Zn^2+^ (or possibly Mg^2+^) has weaker binding with D37, H38 and D136. There is another Zn^2+^ binding site (Cys2His2 zinc finger motif) in a peripheral location of the β-CASP domain, which is not present in SNM1A and SNM1B, other members of the extended structural family of MβL fold enzymes. The C-terminal tail of Artemis is considered as a regulatory domain, autoinhibiting its endonucleolytic activity by interacting with the catalytic domain (20). Residues interacting with a hairpin DNA end are shown, as defined by Liu *et al.* (6). The region of the C-terminal tail of Artemis (three contact points: ∼K368-∼Y407) interacting DNA-PKcs are identified for the basal state in the current study and are indicated by a red double arrow between panels A and B.

**Figure 3.**
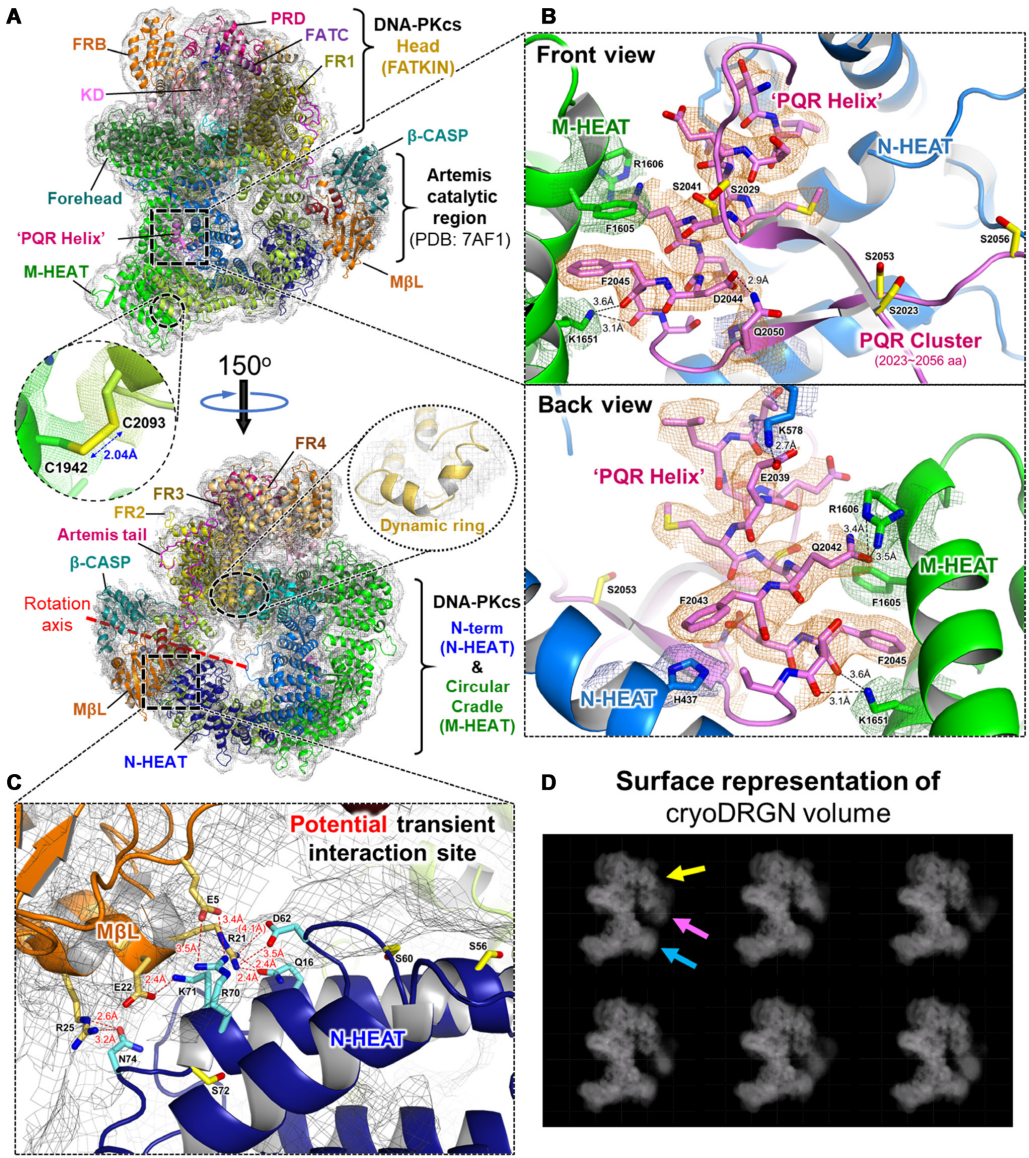
Overall structure of the Artemis:DNA-PKcs complex. (**A**) Overall structure of Artemis:DNA-PKcs, front (top) and, 150°-rotated, back (bottom) views. Blurred map (under EMD-26192, ‘blurred map’) is shown in gray (a *B* factor of +80A^2^, contoured at a level of 3.0). The structure of DNA-PKcs with Artemis tail was deposited to the Protein Data Bank (PDB: 7TYR). The crystal structure of the N-terminal catalytic region of Artemis, manually fit into a potential Artemis density in this figure, consists of 3–361 amino acids (PDB: 7AF1). In this orientation, the catalytic site of Artemis is facing toward DNA-PKcs. An intramolecular disulfide bond (2.04 Å) between C1942 and C2093, which holds two halves of the circular cradle of DNA-PKcs, is shown with a density within the density modified map (1.0 σ) in a dotted circle (under EMD-26192, ‘density modified map’). The connectivity of a dynamic ring of the FR3 (T3198–D3226) was observed in the blurred map. The color scheme is same as in Figure 2. (**B**) Detailed view of the interactions between the PQR helix (T2035–S2046) (magenta) and M-HEAT (green), front (top) and back (bottom) views. The side chain density of the PQR helix and some residues in M-HEAT is shown (1.0 σ) (under EMD-26192, ‘blurred map’). A parallel-displaced π-π interaction by F1605 and F2045 is observed. With K1651, these three residues may form a potential π-π-cation interaction configuration for a tighter intramolecular interaction between the PQR helix and the M-HEAT region. (**C**) Detailed view of the potential transient interaction sites between the N-terminal MβL domain of Artemis and the N-HEAT of DNA-PKcs. Some of these residues may be responsible for weak transient interaction between Artemis and DNA-PKcs such that Artemis resides near the site on DNA-PKcs where Artemis enters upon activation. Serines (S56, S60 and S72) are colored in yellow. Among them, the N site serines (S56 and S72), phosphorylation of which is known to partially inactivate DNA-PKcs activity, are located in this area (colored in yellow). (**D**) Surface representation of cryoDRGN volume. CryoDRGN analysis reveals residual heterogeneity in datasets and dynamic continuous motions of the catalytic domain of Artemis relative to DNA-PKcs. UMAP visualization of the latent space representation of particle images along with select representative density maps is shown in Supplementary Figure S7. Dynamic continuous motions of Artemis catalytic domain relative to DNA-PKcs are shown in Supplementary Videos 4 and 5. It is possible that when the catalytic region of Artemis (magenta arrow) stays bound to the FR2 (yellow arrow), not interacting with the N-HEAT (blue arrow), the dynamic ring of the FR3 (T3198–D3226, shown in below panel A in a zoomed-in view) interacts with Artemis such that the catalytic region is stabilized in this configuration. Readers are referred to the online version of Figure 3D for clearer viewing of the Artemis density in the cryoDRGN images.

Density modification of the state 2 map improved overall interpretation and improved FSC resolution suitable for model building of various features of DNA-PKcs and the C-terminal Artemis sequences bound on DNA-PKcs (Supplementary Figure S2E) (50,51). The atomic model coordinates of DNA-PKcs and a portion of the C-terminal tail of Artemis were deposited to the PDB (PDB: 7TYR). The detailed interactions between the Artemis tail and DNA-PKcs are discussed below. The schematic representation of human DNA-PKcs and the recombinant human Artemis-TEV-(8×)His used in this work is described in Figure 2. Using a blurred map (under EMD-26192, ‘density modified map’), the crystal structure of the N-terminal catalytic domain of Artemis (PDB: 7AF1, 3–361 aa) was manually fit into the Artemis catalytic domain density (Figure 3A). Although a clear density of the ABCDE cluster region is missing in the density modified map, the PQR region is clearly visible. A parallel-displaced π–π interaction by F1605 and F2045 allows the PQR helix (T2035–S2046) to reside on this side of M-HEAT ring (Figure 3B both top and bottom panels). With K1651, three residues may form a π–π–cation configuration for a potentially tighter intramolecular interaction between the PQR helix and the M-HEAT region. In addition, an intramolecular disulfide bond (2.04 Å) between C1942 and C2093, which holds two halves of the circular cradle of DNA-PKcs, is observed (Figures 2A and 3A upper panel, circled insert).

The density presented by the state 2 structure for the catalytic domain of Artemis did not contain clear secondary structural elements but instead consisted of an envelope volume that could contain the first 361 amino acids of Artemis. Four possible orientations were tested with either the MβL domain or the β-CASP domain closer to the N-HEAT repeats of DNA-PKcs, and either the active site of Artemis faces toward or away from DNA-PKcs (Supplementary Figure S6). Among them, the catalytic domain with the MβL domain closest to the N-HEAT repeats of DNA-PKcs with the active site of Artemis facing toward DNA-PKcs had the best apparent fit (Figure 3A). Based on the overall shape of the density compared to the crystal structure of the catalytic domain of Artemis (PDB: 7AF1). This also had the shortest distance (∼27Å gap) between the terminal residue of the catalytic domain (e.g. S361) and the N-terminal end of the C-terminal tail of Artemis that we have modeled as bound to the DNA-PKcs FAT FR2 domain (e.g. S362). CCC values did not show a significant difference between which side the active site was facing, toward or away from DNA-PKcs (CCC = 0.723 or 0.717). However, the low CCC scores are more likely due to the poorly resolved density than atomic coordinate fit (Supplementary Figure S6). In addition, if the active site is facing toward DNA-PKcs, the catalytic domain needs a single rotation of ∼180° along an axis, shown in Figure 3A bottom panel, perpendicular to the direction of the Artemis tail attached to the catalytic domain to reach the DNA end, as seen in the Liu *et al.* structure with activated Artemis engaging the DNA end (6).

In addition to the connectivity of the C-terminal tail of Artemis on the DNA-PKcs FAT domain as displayed in Figure 1C, the blurred map could have potential connectivity between the N-terminal portion of Artemis and some regions of the N-HEAT of DNA-PKcs (Figure 3C and Supplementary Figure S2F). Based on the amino acid sequence of those regions, some polar side chains were identified as potential transient interacting residues (E5, R21, E22 and R25 in Artemis and Q16, D62, R70, K71 and N74 in DNA-PKcs). For example, in the view of Figure 3C, the potential interactions would be (i) Artemis/E5 and DNA-PKcs/R70, (ii) Artemis/R21 and DNA-PKcs/D62 and Q16, (iii) Artemis/E22 and DNA-PKcs/K71 and (iv) Artemis/R25 and DNA-PKcs/N74. A transient interaction site for Artemis as a temporary location before moving inside the HEAT cradle of DNA-PKcs is a likely possibility. This would occur prior to binding the DNA end upon autophosphorylation of DNA-PKcs (6). Interestingly, the N sites (S56 and S72), which are phosphorylated and which partially inactivate DNA-PKcs, are located exactly at this potential interaction site (61). Phosphorylation of these serines may also result in some effect on the interaction with Artemis, or for blocking Artemis from entering inside the HEAT cradle. These considerations compelled us to present such a hypothetical fit of the catalytic domain of Artemis in our maps.

### CryoDRGN reveals a high degree of heterogeneity in datasets and dynamic continuous motions of the Artemis catalytic domain relative to DNA-PKcs

The variable positioning of the Artemis catalytic domain relative to DNA-PKcs limited the obtainable resolution in homogeneous reconstruction. Initial attempts to improve the resolution using RELION multi-body refinement (62,63) and cryoSPARC 3D variability analysis (46) failed, suggesting that Artemis is in continuous motion relative to DNA-PKcs and lacks discrete positioning. To better address this level of continuous motion, we analyzed a select dataset that contained strong Artemis catalytic domain density utilizing the variation autoencoder methodology in cryoDRGN (58). This curated Artemis:DNA-PKcs dataset showing the best Artemis catalytic domain density was used to train the image encoder and volume decoder of cryoDRGN (Supplementary Figure S7A). Reconstructions across the autoencoder latent space comprised of 8D space revealed the Artemis catalytic domain density varied in a continuous arc relative to the FAT and N-HEAT repeat regions (Figure 3D, Supplementary Figure S7B and Supplementary Videos 4 and 5) in addition to the N- and M-HEAT motions relative to one another and FATKIN domains (Supplementary Videos 6 and 7). In reconstructions for specific latent space dimensions, Artemis density becomes well resolved when proximal to the α-helix (FR2_α7: Q3059–H3070), which contains residue L3062 that is mutated in the human SCID mutation in DNA-PKcs, which suggests at least a transient stable interaction with this site (Supplementary Figure S7B; numbers 1 & 2, Supplementary Figure S7C and Supplementary Video 5). Additional contacts appear in reconstructions with the N-HEAT domain that is displaced upon DNA binding to DNA-PKcs (Supplementary Figure S7B; number 3). In addition to the improved understanding of Artemis catalytic domain positioning, density attributable to DNA-PKcs in the DNA binding region of DNA-PKcs was also shown to be highly variable but stable in some complex configurations, supporting the PQR resolution in our state 2 map.

It is possible that when the catalytic region of Artemis stays bound to the FR2, not interacting with the N-HEAT as shown in the Supplementary Figure S7C, the dynamic ring of the FR3 (T3198–D3226), which is observed in the blurred map, (Figure 3A bottom panel, circled insert) may interact with Artemis such that the catalytic region can continue to reside there (Figure 3D). This feature was more prominent in 3D variable analysis of Artemis:DNA-PKcs state 1 (Supplementary Figure S2G). As the forehead and jaw open up (by 10–13Å), the catalytic domain of Artemis appears, and then the distance between the dynamic ring of DNA-PKcs and the Artemis catalytic domain becomes shorter. This configuration coincides with clearer density for the Artemis tail on DNA-PKcs and density for the dynamic ring in the FR3 subdomain associated with the catalytic domain of Artemis (Supplementary Video 2).

### Interaction between DNA-PKcs and the C-terminal regulatory region of Artemis

**Figure 4.**
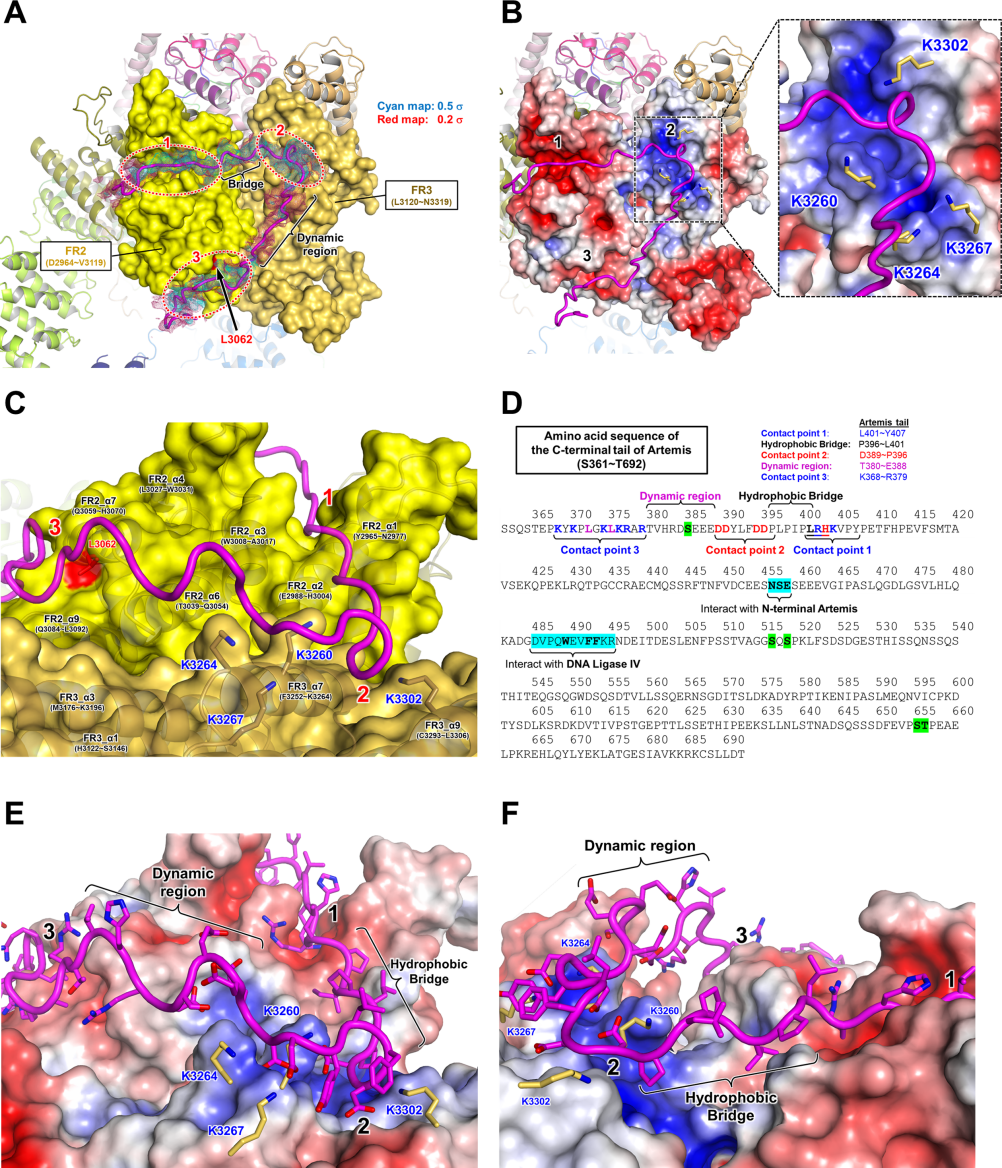
Three contact points on DNA-PKcs for the C-terminal regulatory tail of Artemis. (**A**) Three contact points on DNA-PKcs with the Artemis C-terminal tail. FR2 (yellow) and FR3 (dark yellow) of DNA-PKcs are shown in surface representation. The density corresponding to the Artemis tail from the density modified map is shown in cyan (0.5 σ) and red (0.2 σ) (under EMD-26192, ‘density modified map’) and the backbone of the Artemis tail is shown as a magenta tube (PDB: 7TYR). Three contact points are circled with red dotted lines numbered 1, 2 and 3. The bridge region between the contact points 1 and 2 and the dynamic region of the tail with poor density are also labeled. L3062 in FR2 subdomain is colored in red. Note that patients with the L3062R mutation in DNA-PKcs are known to develop radiosensitive SCID with insufficient Artemis activation and reduced end-joining activity. (**B**) Surface charge distribution of the FR2 and FR3 subdomains. The electrostatics calculation was based on APBS (Adaptive Poisson-Boltzmann Solver) in PyMOL. Red and blue colors represent negatively charged region (e.g. contact points 1 and 3) and positively charged region (e.g. contact point 2), respectively. A turn of the Artemis tail winds through a four-lysine network (K3260, K3264, K3276 and K3302) of the FR3 subdomain. A zoomed view of this 4-K network is shown. The detailed interactions between these lysines and the Artemis tail are described in Figure 5C. (**C**) Different view of three contact points with the Artemis tail. Three contact points are again labeled with red numbers. Some helices of FR2 and FR3 domains are shown in cartoon representation. The side chain of the four lysines (K3260, K3264, K3276 and K3302) is shown. L3062 is colored in red. (**D**) Amino acid sequence of the C-terminal tail of Artemis (361–692 aa). The tail regions which interact with DNA-PKcs are labeled with colors as described (368–407 aa). The tail region which interacts with contact point 1 (L401–Y407) contains positively charged residues including H403. The region which interacts with contact point 2 (D389–P396) contains negatively charged residues. The region which interacts with contact point 3 (K368–R379) is highly positively charged. The hydrophobic bridge (P396–L401) is located between contact points 1 and 2. The dynamic region (T380–E388) also contains many polar and charged residues, including S385 which is known to be a basal phosphorylated serine. The Ser and Thr residues highlighted in green are known to be phosphorylated residues in the basal state. (**E** and **F**) Different views of three contact points with the Artemis tail. A surface charge distribution of the FR2 and FR3 domains is shown. The side chains of the Artemis tail are shown. The backbone of the tail is shown in a magenta tube representation.

The strong visual evidence of the position of the catalytic domain of Artemis also gave insight into the direction of the C-terminal tail of Artemis on the FAT domain of DNA-PKcs. Based on both the density modified map and blurred map, three distinct contact points between the C-terminal tail of Artemis and the FR2 and FR3 subdomains of the FAT can be identified as the Artemis residues just C-terminal of the catalytic domain are following along the inter-helical interfaces of the FAT domain (Figures 2 and 4A and Supplementary Figure S8A). A nearly identical path of the Artemis extended tail was also seen in Liu *et al.*’s recent Artemis:DNA-PKcs:Ku70/80:DNA structure (PDB: 7SGL), but no detailed assessment on those interactions was discussed (6). In our positioning of amino acids, a slightly different set of residues is bound to the FR2 and FR3 subdomains of DNA-PKcs FAT domain from Liu *et al.* (Supplementary Figure S8B). Due to a low quality of some regions of our Artemis density, the validity of the positioning of amino acids of the tail still holds uncertainty, and one can try to register other amino acids in the same path with reasonable fitting. It is possible that the register of the amino acids can be variable, and the Artemis tail might slide back and forth on the DNA-PKcs FAT domain's helical grooves. Given the dynamic positioning of the N-terminal catalytic domain of Artemis, it seems plausible the C-terminal tail of Artemis adopts variable interactions on the FAT domain. This loose interaction of the tail might contribute to the catalytic region's continuous interaction mode external to the N-HEAT repeats of DNA-PKcs and when positioned internal to the HEAT cradle to engage the DNA end (Supplementary Figure S8B).

**Figure 5.**
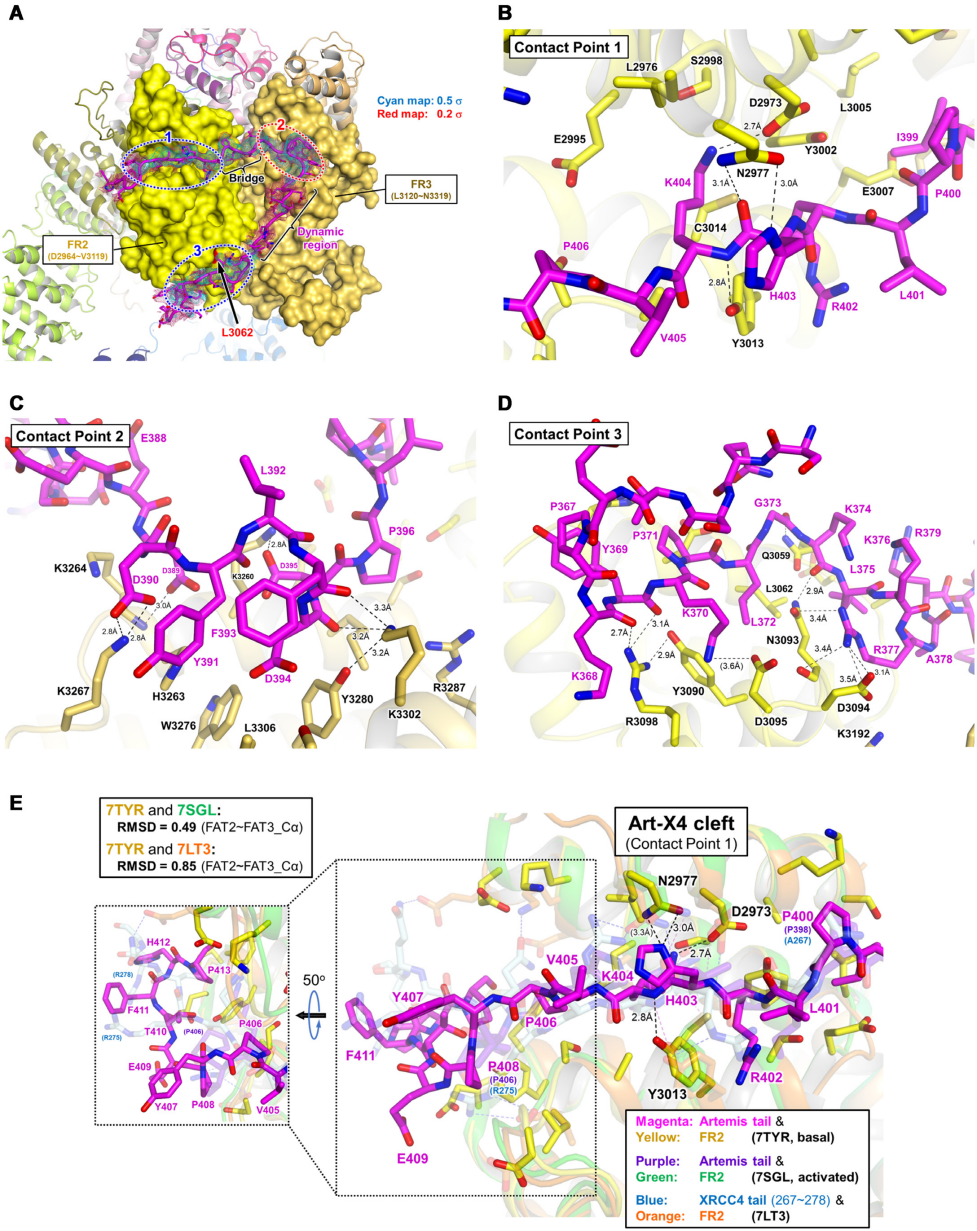
Proposed interaction between DNA-PKcs and the C-terminal regulatory tail of Artemis. (**A**) The density of Artemis tail with side chains on the FAT domain. The tail regions which interact with the contact points 1 and 3 are circled with blue dotted lines which represent positively charged sites (PDB: 7TYR). The tail which binds the contact point 2 is circled with a red dotted line, which represents a negatively charged region. See the Figure 4D for the detailed explanation. (B–D) Analysis of potential interactions in the contact point 1 (**B**), contact point 2 (**C**) and contact point (**D**) without density maps. The Artemis tail is shown in magenta. Possible interactions are indicated with dashed lines. The same figures with the density map are shown in Supplementary Figure S8. (**E**) Overlaid Artemis tails (basal and activated states) and XRCC4 tail in the Art-X4 cleft. The FR2 and FR3 subdomains of three models (PDB: 7TYR, 7SGL and 7LT3) were aligned. The Artemis tail and the FR2 of the basal state of Artemis:DNA-PKcs (7TYR, basal) are shown in magenta and yellow, respectively. The Artemis tail and the FR2 of the activated state of Artemis:DNA-PKcs:Ku:DNA (7SGL, activated) are shown in purple and green, respectively. The XRCC4 tail and the FR2 of the long-range synaptic complex (7LT3) are shown in blue and orange, respectively. 7SGL and 7LT3 as well as all FR2 cartoons are dimmed. The detailed interaction of XRCC4 tail and the FR2 is shown in Supplementary Figure S9C.

Potential interaction sites observed within our complex are highlighted in detail (Figures 2, 4 and 5). Specifically, contact point 1 is located in the FR2 (∼2966–∼3020 aa). Contact point 2 is located in the FR3 (∼3252–∼3309 aa). Contact point 3 is located in the FR2 (∼3057–∼3098 aa). There is a bridge region between contact points 1 and 2 with a clear density but with no contact with DNA-PKcs (Figure 4A). The density of the region between contact points 2 and 3 is poor, indicating that this dynamic part of the tail has fewer contacts with DNA-PKcs; and this would allow flexibility and possible floating of this portion of the tail, without interacting with the DNA-PKcs surface (Figure 4A).

Surface charge distribution was assessed from the electrostatics calculation based on APBS (Adaptive Poisson-Boltzmann Solver) in PyMOL, labeling red for negatively charged and blue for positively charged. The surface of contact point 1 of DNA-PKcs is negatively charged (Figure 4B) and the Artemis tail (L401–Y407) appears buried well into the groove between the FR2_α1 (Y2965–N2977) and the FR2_α2 (E2988–H3004) (Figure 4C and D). H403, known to interact with DNA-PKcs (64), is located in this region, potentially contributing hydrogen bonding with N2977 (Figure 5B and Supplementary Figure S8C). Strikingly, an extra density was observed at the same site as contact point 1 in Chen *et al.*’s 4.60-Å long-range synaptic complex (EMD-23511, later improved to 4.1 Å), where they placed part of the C-terminal tail of XRCC4 (A267–R278), based on the electrostatic surface (PDB: 7LT3) (32) (Supplementary Figures S9A–C). The maps of the XLF-mediated dimer at 4.14 Å (EMD-12299) and monomer at 4.29 Å (EMD-12301) reported in Chaplin *et al.* also contain this extra density (Supplementary Figure S9D). However, they neither registered any amino acids in this density (PDB: 7NFC and 7NFE) nor discussed the source of this density (34). Based on our experience, it is unlikely that their DNA-PKcs preparations contained Artemis. If this density is not Artemis in these maps but rather XRCC4 C-terminal residues, this suggests that Artemis would need to be displaced in the progression of DNA-PKcs complexes leading toward end-ligation during the NHEJ process.

We propose calling this region where the Artemis tail (L401–Y407) and XRCC4 tail (A267–R278) are proposed to bind, which encompasses the FR2 subdomain of the FAT domain of DNA-PKcs (∼2966–∼3020 aa), the ‘Art-X4 cleft’ (Figure 2A). This cleft consists mainly of three α-helices (FR2_α1: Y2965–N2977; FR2_α2: E2988–H3004; FR2_α3: W3008–A3017) and a loop region (S3018–Y3036) with the side of the cleft expanding to residue 3036, and they contribute to a highly negatively charged cleft (Figure 4B, number 1). Two helices, FR2_α1 and FR2_α3, are responsible for Artemis interaction. Based on the assigned amino acids, interactions between the Artemis tail or the XRCC4 tail with the Art-X4 cleft appear relatively weak (Figure 5B and E and Supplementary Figures S8C and S9C). Thus, this loose interaction in this cleft may explain why either of two different proteins can bind at the same cleft. Therefore, this potential mutually exclusive binding site for Artemis and XRCC4, and potentially for other proteins, may provide a broad protein-protein interaction region, which may be responsible not only for providing the platform for NHEJ components but also for allowing the NHEJ mechanism to enforce a processing step (Artemis nucleolytic action) prior to ligation by the XRCC4:ligase IV complex. Among ∼24 residues located on the surface of the cleft, there are four residues (i.e. L2976, Y3002, L3005 and Y3013) conserved among jawed vertebrates (11). Y3013 is one of the DNA-PKcs residues interacting with our basal state Artemis tail (Figure 5B). Interestingly, S3018, one of the surface cleft residues, but not interacting with Artemis, is unique to DNA-PKcs among jawed vertebrates. Other relevant residues on the cleft surface are also highly conserved (e.g. K2991, E3007, C3014, I3019, P3034, F3035 and Y3036). Comparison of the Artemis tail at contact point 1 between the basal state and the activated state shows that the tail shifts to outside of the cleft away from the FR2 (Supplementary Figure S8B) (6). Both structures show that N2977 and D2973 are the key residues that interact with the Artemis tail (Figure 5B and Supplementary Figures S8C and S8D). Interestingly, both N2977 and D2973 residues also interact with the XRCC4 tail (Supplementary Figure S9C) (32). The role of this Art-X4 cleft is still unknown, but there is no known disease mutation in this cleft or in this portion of Artemis (1). The R275X mutation (X denotes a stop codon) in XRCC4, known to cause short stature and microcephaly, may have weaker binding with DNA-PKcs because Q277 of XRCC4 appears to interact with E2988 of DNA-PKcs in a long-range synaptic complex (Supplementary Figure S9C) (32).

**Figure 6.**
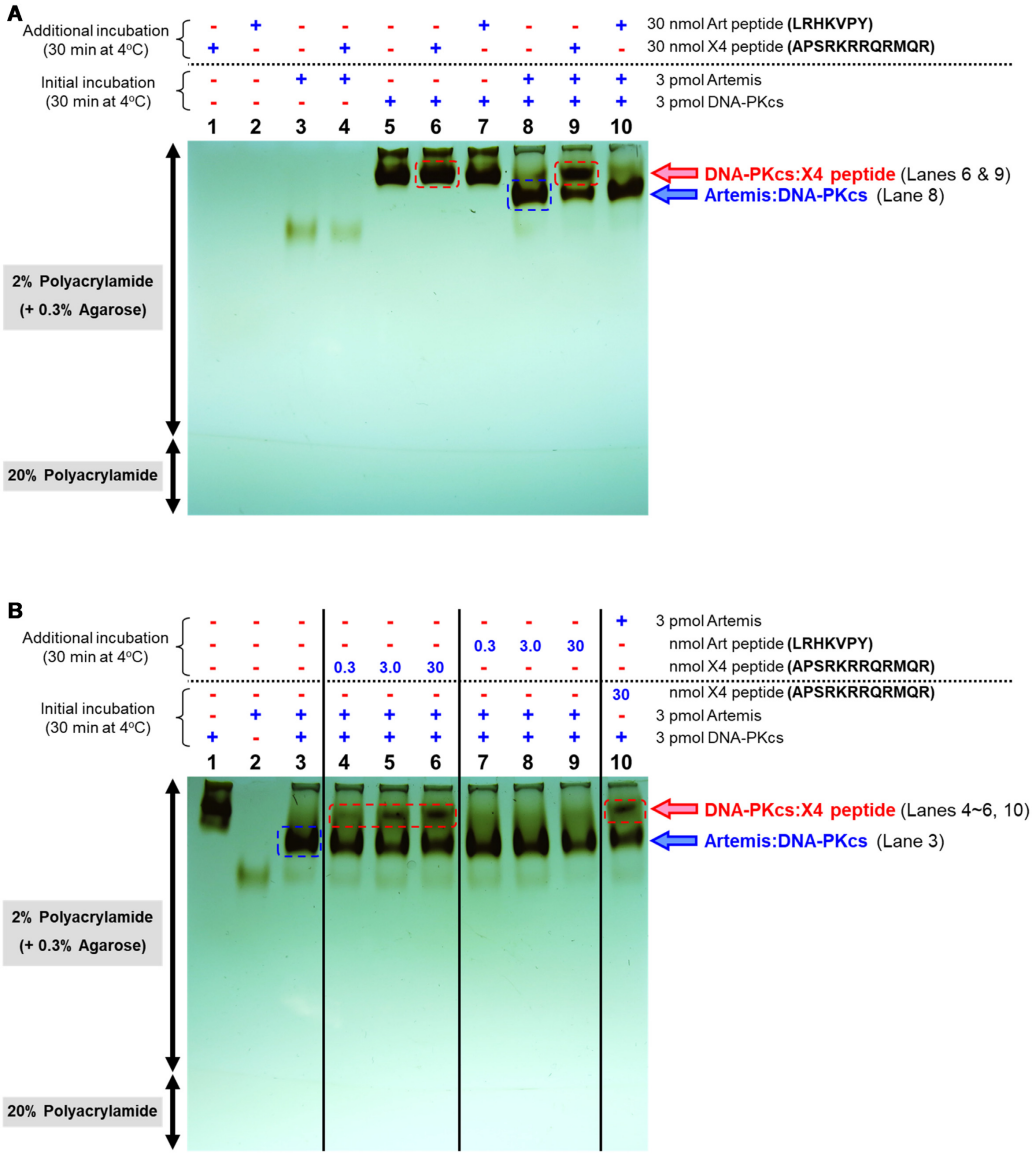
Competition assays using the XRCC4 peptide on the Artemis:DNA-PKcs complex. (**A**) The effect of addition of the XRCC4 peptide or Artemis peptide on the Artemis:DNA-PKcs complex. The binding reaction was carried out as described in MATERIALS AND METHODS and Figure 1A. Two peptides, XRCC4 peptide (X4: APSRKRRQRMQR, corresponding to A267-R278) and Artemis peptide (Art: LRHKVPY, corresponding to L401–Y407) were chosen based on Chen *et al.* (32) and our work here, respectively. Both peptides are considered to bind to the Art-X4 cleft. Ten thousand-fold more peptide (e.g. 3 mM) was used to test the effect on the addition of peptides to each component including the Artemis:DNA-PKcs complex. In order to test whether these peptides disrupt the complex formation, they were added into the pre-formed Artemis:DNA-PKcs complex and incubated at 4°C for another 30 min (lanes 9 and 10). The samples were run on an agarose-acrylamide composite native gel, composed of 2.0% polyacrylamide and 0.3% agarose, at 4°C. The gel was stained with silver nitrate. The complexes of Artemis:DNA-PKcs and the potential DNA-PKcs:XRCC4 peptide are highlighted in blue and red, respectively. (**B**) The effect of various concentrations of peptides on the Artemis:DNA-PKcs complex. Various concentrations of each peptide were used (e.g. 30 μM, 300 μM and 3 mM) as indicated. The X4 peptide or Art peptide was added into pre-formed Artemis:DNA-PKcs complex (lanes 4–6 and 7–9, respectively). Also, DNA-PKcs was incubated with the X4 peptide first and then Artemis was added (lane 10) to test the effect of the order of addition. The same gel system was used as in panel (A).

To address this hypothesis of mutually exclusive binding between XRCC4 and Artemis, competition assays using two peptides in the agarose-acrylamide gel were performed. XRCC4 peptide (X4: APSRKRRQRMQR) was chosen based on Chen *et al.* (32) and Artemis peptide (Art: LRHKVPY) was designed based on our basal state structure. These peptides are highly positively charged (theoretical pI of 12.60 and 9.99, respectively) and are not likely to enter the gel in the 1× TBE (pH ∼8.3) condition (Figure 6A, lanes 1 and 2). No apparent shift was observed upon the addition of X4 peptide to Artemis (Figure 6A, lane 4) and the addition of X4 peptide or Art peptide to DNA-PKcs (Figure 6A, lanes 6 and 7). Importantly, the addition of X4 peptide, but not Art peptide, to Artemis:DNA-PKcs complex disrupted its complex formation, dissociating Artemis from DNA-PKcs (Figure 6A, lanes 9 and 10). The addition of Art peptide may have a small disruptive effect on the complex formation but it is limited and similar to the control lane (Figure 6A lanes 8 and 10). Various concentrations of the peptides were also tested (Figure 6B). While Art peptide might show the reduction of Artemis:DNA-PKcs complex only at high concentrations of Artemis peptide (e.g. 10,000× more) (Figure 6B, lanes 7–9), X4 peptide shows a clear disruption effect in a concentration dependent manner (Figure 6B, lanes 4–6). As expected, addition of Artemis to the pre-incubated DNA-PKcs and X4 peptide resulted in a band at the same position as DNA-PKcs alone, likely due to the DNA-PKcs:X4 peptide complex, indicating that the presence of X4 peptide prevents Artemis from binding to DNA-PKcs (Figure 6B, lane 10).

However, even if these are mutually exclusive sites, because there are quite a number of interaction regions between the C-terminal tail of Artemis and the FAT domain of DNA-PKcs and because there could be potentially other unidentified interaction sites between XRCC4 and DNA-PKcs, such competition assays with peptides may not reflect the complete mechanism. For example, the positively charged XRCC4 peptide still may bind to contact point 3, which is another negatively charged area and in fact more critical for Artemis catalytic domain positioning prior to its transitioning to DNA ends. Therefore, this disruptive effect may be due to the binding of XRCC4 peptide to contact point 3, in addition to contact point 1. More systematic tests such as pull-down experiments will be required to define this precisely.

Since XRCC4 is complexed with DNA ligase IV, and DNA ligase IV interacts with Artemis, the interpretation of the data is likely more complicated in a gel shift assay and even in biophysical experiments using those purified proteins in attempts to elucidate the true mechanism. Nevertheless, these competition assays show that the C-terminal portion of XRCC4 (A267–R278) disrupts Artemis:DNA-PKcs complex and that it also prevents the Artemis:DNA-PKcs complex formation. The findings are significant as DNA-PKcs may recruit XRCC4:Ligase IV complex to broken DNA sites.

Our previous study shows that C-terminally deleted XRCC4 neither affects DNA ligase IV binding and activity, nor the in vivo function of XRCC4 (65). Thus, the C-terminal tail of XRCC4 is not necessary for XRCC4:DNA ligase IV to ligate the broken DNA ends. However, one could imagine that the transition from the Artemis:DNA-PKcs complex to the DNA-PKcs:XRCC4:DNA ligase IV complex at the broken DNA ends may become less efficient when the XRCC4 tail is absent. In addition, interactions of XRCC4 with the other NHEJ components involve both head group and tail interactions of XRCC4 with other components, including Ku and DNA-PKcs, in addition to interaction with ligase IV (1,66–68).

Contact point 2 occurs between positively charged residues of the FR3 domain (∼3252–∼3309 aa) and Artemis residues (D389–P396) (Figures 2, 4B, number 2, and 4D) and includes a sharp bend down toward the catalytic domain. Stabilizing this turn in the Artemis tail is a four-lysine network (K3260, K3264, K3276 and K3302) (Figures 4B zoomed view and 4C) and these four lysines also interact with the tail (Figure 5C and Supplementary Figure S8E). Each of two sides of that quadrangle are adjacent to the border between contact point 2 and the hydrophobic bridge and between contact point 2 and the dynamic region of the tail, respectively (Figure 4E and F). For our basal state complex, K3260 stands at the turning point of the tail as physical support and the tail goes off to the dynamic region through K3260 and K3264. In the equally plausible structure from Liu *et al.* for the activated state, which shows a similar turn and location of the Artemis tail, the Artemis tail seems to run slightly over these two lysine residues and does not run between them as our density suggests (Supplementary Figure S8F) (6).

Hydrophobic residues (P396–L401) were assigned to the bridge region between the contact points 1 and 2 as the hydrophobic bridge with no contact with DNA-PKcs (Figure 4D–F). As for the dynamic region between the contact points 2 and 3, a stretch of highly charged and polar residues (T380–E388) was assigned (Figure 4D–F). This includes S385, which is known to be a basal state phosphorylated serine (69).

### Contact point 3 and analysis of a hypomorphic L3062R mutation of DNA-PKcs

**Figure 7.**
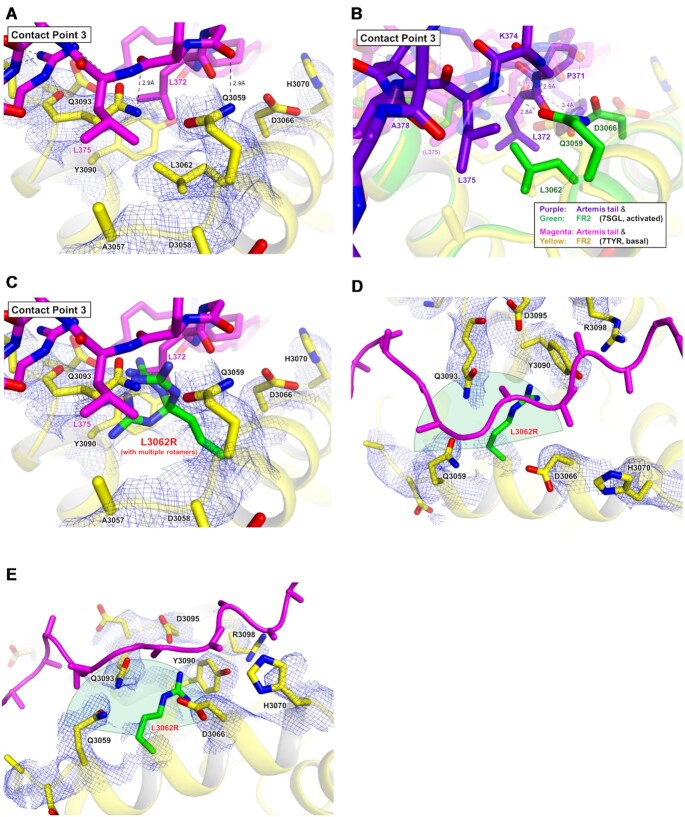
Structural analysis of the L3062R mutation of DNA-PKcs. (**A**) The local environment around the L3062 residue in contact point 3. L3062 of the FR2_α7 (Q3059-H3070) is located in contact point 3 and contributes a local hydrophobic environment with the L372 and L375 residues of Artemis. (**B**) Overlaid Artemis tails (basal and activated states) in contact point 3, highlighting the activated state. The Artemis tail and the FR2 of the activated state of Artemis:DNA-PKcs:Ku:DNA (PDB: 7SGL) are shown in purple and green, respectively. The Artemis tail and the FR2 of the basal state of Artemis:DNA-PKcs (PDB: 7TYR) are shown in magenta and yellow, respectively. 7TYR and both FR2 cartoons are dimmed. (**C**) Substitution with the hypomorphic L3062R mutation with multiple rotamers. Only three rotamers are shown. Patients with the L3062R mutation in DNA-PKcs are known to develop radiosensitive SCID with insufficient Artemis activation and reduced DNA end joining activity. The side chain of L3062R not only would directly interfere with the Artemis tail due to its close proximity but also could interfere with some residues of DNA-PKcs, including Q3093, Y3090, Q3059 and D3066, potentially altering the overall interaction with Artemis. (**D** and **E**) Different views of one of L3062R rotamers and its environment. A green sector is shown to demonstrate how L3062R can overlap with other residues.

Contact point 3 is another negatively charged region in the FR2 region of the FAT domain (∼3057–∼3098 aa) and the positively charged residues (K368–R379) of Artemis interact with the surface of this region (Figures 2, 4B, D and 5D and Supplementary Figure S8G). L3062 of the FR2_α7 (Q3059-H3070) is located in this contact point 3 and contributes a local hydrophobic environment with the L372 and L375 residues of Artemis (Figure 7A). Patients with a L3062R mutation in DNA-PKcs are known to develop radiosensitive SCID with insufficient Artemis activation and reduced end-joining activity (70). It is important to note that only one of three contact sites for Artemis is disrupted by L3062R to cause this disease. Because L3062R DNA-PKcs is known to retain its kinase activity, its overall folding may not be largely disrupted (70). The recent Liu *et al.* activated state Artemis:DNA-PKcs:Ku70/80:DNA structure also shows a similar positioning of the Artemis tail relative to DNA-PKcs where L3062 of DNA-PKcs is sandwiched between L372 and L375 of the Artemis tail (Figure 7B) (6). However, the side chains of L372 and L375 of the activated state Artemis tail are positioned closer to the side chain of L3062 of the FR2 subdomain compared to the position of the basal state, as a hydrogen bond between the oxygen of the Q3059 side chain and the nitrogen of the L375 backbone form, providing additional stability. This would suggest that the catalytic domain of Artemis, which is located in the circular cradle, is partially pulling the tail along with it.

In-silico mutagenesis analysis of our model shows that the side chain of L3062R not only would directly interfere with the Artemis tail due to its close proximity but also could interfere with residues of DNA-PKcs, Q3093, Y3090, Q3059 and D3066, potentially altering the overall interaction with the Artemis tail as well as the local FAT domain tertiary structure (Figure 7C–E). Moreover, K374 of Artemis and the L3062R mutation in DNA-PKcs may have a repulsive effect. It is important to note that other interacting residues around L3062 within the contact point 3 seem sufficient to stabilize the Artemis:DNA-PKcs complex, even if L3062 is mutated to arginine. This agrees with recent work showing that the L3062R mutation in full-length DNA-PKcs reduced the interaction with Artemis by 5- to 10-fold, but the interaction was not completely inhibited (39). This also agrees with the observation that the L3062R mutant of DNA-PKcs can still recruit Artemis to the DSB sites, but leads to insufficient Artemis activation, resulting in disruption of coding joint formation (70). Therefore, it is likely that this L3062R mutation makes it more difficult for the Artemis catalytic domain to interact with the N-HEAT repeats, and hence, prevents the catalytic domain from relocating to the DNA end, resulting in reduced nuclease activity (6).

### The highly dynamic nature of the C-terminal regulatory region of Artemis

Purification of recombinant Artemis results in a symmetrical peak from a size exclusion column and no severe aggregation or precipitation was observed from the purified Artemis (Supplementary Figure S1B). The elution profile corresponds to a dimeric size (∼160 kDa). Such a large hydrodynamic radius of Artemis could be due to the dynamic nature of its extended C-terminal tail. Alternatively, an extended C-terminal region might interact with the size exclusion resin to run slower. Therefore, this size exclusion chromatography profile does not reflect the molecular size of Artemis. During a registration step of amino acids of the Artemis tail, we also utilized RoseTTAFold, a tool that uses deep learning to predict protein structure, to compute the structure of the C-terminal tail of Artemis. The result revealed some helices in the tail, but the overall tail is relatively free of tertiary structural folds driven by secondary elements and is predicted to be extended, suggesting a dynamic strand around the catalytic domain of Artemis (Supplementary Figure S10A and Supplementary Video 8) (59). The Artemis structure predicted by AlphaFold v2.0 is nearly devoid of secondary structure and devoid of higher folding (Supplementary Figure S10B) (60,71). A part of the predicted C-terminal tail seems to locate near the catalytic region (Supplementary Figure S10C). Now with two structural studies of Artemis bound to DNA-PKcs (this study and Liu *et al.*, 2022), the C-terminal regulatory region of Artemis remains mostly unresolved with ∼280 amino acids still unaccounted for, and this region could interact with other components of the NHEJ complex or with histones on the DNA ends (Figure 2B) (72,73). This unresolved tail extending from the Art-X4 cleft may interact with the Artemis catalytic domain to allow the Artemis to stay bound to the FAT domain more tightly (Supplementary Figure S5D). This intramolecular interaction between the catalytic domain (S2–Y11) and the tail (N456–E458) could account for autoinhibition of Artemis (Figure 2B) (20). Importantly, the role of phosphorylation of the C-terminal tail of Artemis is still unknown. Such a long unstructured tail would be very challenging to predict by AlphaFold, which currently provides only a single state.

### Possible transition from the basal state to the activated state of Artemis:DNA-PKcs

**Figure 8.**
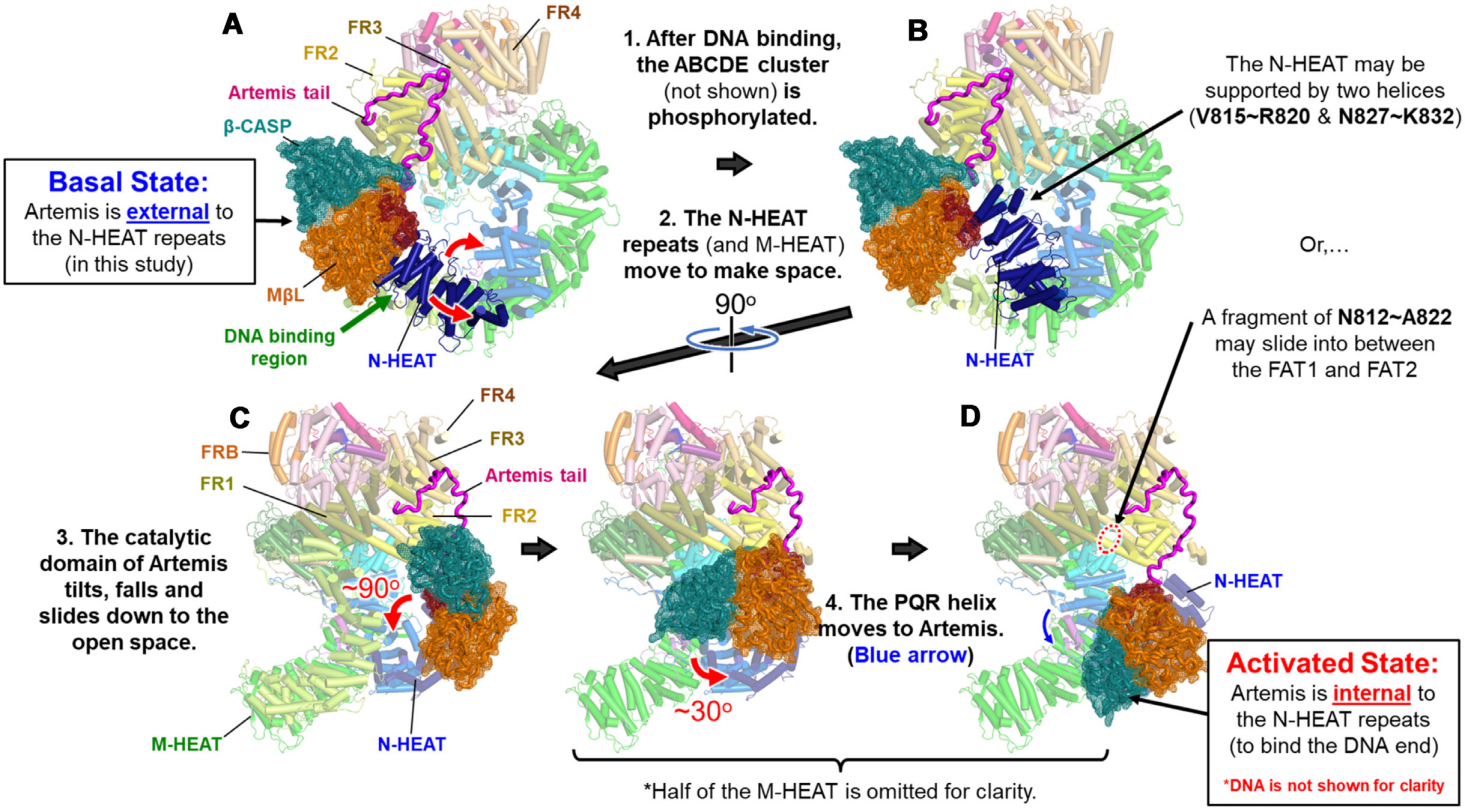
Possible transition from the basal state to the activated state of Artemis:DNA-PKcs. A potential transition model of the catalytic domain of Artemis from the basal state to the activated state is described. The color scheme of proteins is the same as in Figure 2. The PDB: 7TYR (this study) and PDB: 7AF1 (crystal structure of Artemis catalytic domain) are shown in cylinder and tube representations, respectively (**A**). The formation of the Artemis:DNA-PKcs complex may reflect the basal state in which the catalytic domain of Artemis remains external to the N-HEAT repeats, as seen in this study (A). The Artemis:DNA-PKcs:DNA complex without incorporation of ATP/Mg^2+^ may be considered to be a pre-activated state (not shown in this figure for clarity). Once Artemis:DNA-PKcs binds a DNA end and autophosphorylation of the ABCDE occurs in the presence of ATP/Mg^2+^, the N-HEAT repeats, and perhaps M-HEAT repeats, undergo conformational changes to make space between the N-HEAT and M-HEAT repeats (**B**). This allows the Artemis catalytic domain to relocate into that open space to access the DNA end while the C-terminal tail remains tethered on DNA-PKcs (**C**). Then, the PQR helix, which stays bound on the M-HEAT region, moves close to Artemis (**D**) (6). In the DNA-PK complex (PDB: 7LT3), two helices of DNA-PKcs (V815–R820 and N827–K832) bind the internal part of the N-HEAT region above the dsDNA (32). In contrast, one of these fragments, N812–A822, is found between the FR1 and FR2 in the activated Artemis:DNA-PKcs:Ku70/80:DNA (6). It is not clear when this transition occurs.

Our cryo-EM structures of the Artemis:DNA-PKcs complex and Artemis:DNA-PKcs:DNA complex show that while the Artemis tail is bound on the FAT domain, the N-terminal catalytic region of Artemis remains external to the HEAT cradle of DNA-PKcs and thus restricted from the DNA end. In the activated complex, the opening between the N-HEAT repeats and the FAT domain requires large rearrangements to the HEAT domains to allow Artemis to enter between the N- and M-HEAT rings, as described by Liu *et al.* (6). Initial complex formation between DNA-PKcs and Ku70/80 bound to DNA similarly has a HEAT cradle configuration that would restrict Artemis access to the DNA end as the Ku70 and a FATKIN shifted N-HEAT repeat would protect the DNA end from Artemis access. Thus, in order for Artemis to reach the DNA end, DNA-PKcs must undergo extensive conformational change to make space for Artemis, and the catalytic domain of Artemis must move into that space while the C-terminal tail remains tethered at the Art-X4 cleft of DNA-PKcs (6) (Figure 8). However, there are likely a number of fine intermediate conformations between the basal state and the activated state. The DNA end structure (5′ or 3′ overhang versus hairpin) likely impacts the overall assembly and order of steps in the NHEJ process (6). It is unknown whether a DNA end with an overhang behaves like a hairpin DNA end such that it promotes *in-cis* autophosphorylation or whether it favors the protection mode.

One series of steps by which the catalytic domain of Artemis might translocate from a position external to the DNA-PKcs N-HEAT ring, as it is in the basal state, to an internal position to contact the DNA end, is diagrammed in Figure 8. Once the Artemis:DNA-PKcs complex binds the DNA end through the DNA-PKcs N-HEAT repeats, it would undergo *in-cis* autophosphorylation of the ABCDE cluster, which will change the position and conformation inside the circular cradle ring of DNA-PKcs along with both the N- and M-HEAT rings. For example, the N-HEAT ring may move slightly to the upper right and radially outward to make space. This N-HEAT (parts of L29–L44 and R82–M96) may be supported by two helices (V815–R820 and N827–K832), which can be seen between the N-HEAT and DNA in a DNA-PK structure in a synaptic complex (PDB: 7LT3) (32). Interestingly, this density was not seen near the N-HEAT at all in the Artemis:DNA-PKcs:Ku70/80:DNA structure (PDB: 7SGL). N812–A822 was, instead, placed between the FR1 and FR2, as if it locks the movement of the FAT domain, although the density of the fragment is not clear (6). In our Artemis:DNA-PKcs map, those two helices are not clearly visible but a focal density which may correspond to them was observed at the bottom of the FR1 and FR2. Moreover, at some point, the DEB-A and DEB helices (2724–2767 aa) in the middle of DNA-PKcs may move away. Then, once the space is created, the catalytic domain of Artemis tilts, falls and slides down to it so that the position of the β-CASP is located at the center of the N- and M-HEAT rings. Then, the PQR helix (T2035–S2046) moves from the M-HEAT region to the Artemis side as seen in the Artemis:DNA-PKcs:Ku70/80:DNA complex (PDB: 7SGL) (6). This overall mechanism could be similar in the absence of or in the presence of Ku70/80. However, it is expected that more movement would be needed in the presence of Ku70/80. It is not known whether Artemis remains bound and juxtapositioned to Ku and the N-HEAT repeats prior to the conformational change of DNA-PKcs. Our analysis shows that binding of DNA-PKcs to DNA did not trigger the transition of the Artemis catalytic region. Therefore, even without Ku70/80, it is likely that the ABCDE phosphorylation is the next step in the mechanism for Artemis to move to the DNA end. Clearly, a large conformational change between the N-HEAT and the M-HEAT regions with or without Ku70/80 must occur for Artemis to access the DNA end.

Since Artemis can interact with the catalytic domain of DNA ligase IV, it is possible that the Artemis:DNA-PKcs complex may also bring DNA ligase IV to the DNA end through the C-terminal tail of Artemis (D485-R495) (35,36,72). However, it is still unclear how the Artemis:DNA-PKcs complex dissociates from the processed DNA bound by Ku70/80 prior to a ligation event by XRCC4:DNA ligase IV.

## CONCLUDING COMMENTS

In summary, our efforts have been focused on the basal state of the Artemis:DNA-PKcs endonuclease complex because this is the majority state of both Artemis and DNA-PKcs in a cell without DNA damage and even in a cell with DNA damage, since only a small percentage of the 50,000–70,000 molecules of DNA-PKcs and Artemis in each human nucleus become activated. Thus, this is the form that would need to be blocked from activation by an intended inhibitor. We provide structural insight into the basal state of the Artemis:DNA-PKcs interaction and show how dynamic the catalytic domain of Artemis can be. We also describe the Artemis:DNA-PKcs:DNA complex, which is similar to the pre-activated state where DNA-PKcs is bound on DNA, but the phosphate-transfer reaction by the kinase has not yet begun, given the absence of ATP. Our improved agarose-acrylamide composite gel shift assay has the capability of resolving large NHEJ multicomplexes without crosslinking, including Artemis:DNA-PKcs (∼547 kDa) and DNA-PKcs:Ku70/80:DNA (e.g. >∼650 kDa), and the potential for other types of analysis. We utilize this gel system to demonstrate that an XRCC4 peptide, which supposedly binds to the Art-X4 cleft, competes with Artemis and disrupts Artemis:DNA-PKcs complex formation.

Recently, the Blundell group reported cryo-EM structures of DNA-PKcs with ATPγS and various inhibitors (wortmannin, NU7441, AZD7648 and M3814) including drug candidates undergoing clinical trials, providing insights into the mode of PIKK regulatory domain (PRD) upon ligand binding (74,75). Targeting the Artemis active site to inhibit its endonuclease activity has been ongoing by various groups (37,38). In addition to the attempt to develop selective inhibitors for enzymes, protein-protein interaction sites are also potential targets for small molecules or peptides to disrupt the interactions (76,77). For example, peptides that consist of interaction residues of Artemis to DNA ligase IV significantly enhanced the radiosensitivity of some cell lines and, with irradiation, showing synergistic effects as a radiosensitizer in gallbladder cancer and cervical cancer cells (78). Our competition assays also confirmed a potential inhibitory peptide sequence which can be improved further via rational drug design or mRNA display technology (79). Therefore, targeting Artemis:DNA-PKcs interaction sites might be considered for potential drug design.

If one could block the assembly of the Artemis:DNA-PKcs complex, then this would reduce the chance of Artemis conversion to an active form. Inhibition of activation of Artemis in acute lymphoblastic leukemia (ALL) cells would result in chromosomal breaks because the large majority of ALL tumors have ongoing expression of RAG1/2. Even after such pre-B or pre-T cells have exhausted their endogenous V, D and J segments, off-target action by RAG1/2 has been well documented in such cells (80). Thus, Artemis inhibition would generate chromosomal breaks at multiple locations in the genome due to failure to open the DNA hairpins that are generated by RAG1/2. The advantage of this approach is that absence of Artemis has no clinical consequences in nonlymphoid cells unless patients are given radiation therapy or topoisomerase II inhibitors, indicating that there would be no side effects of such inhibitors. Also, Artemis inhibitors would not affect memory B and T cells in the patients, thus largely preserving their existing immunity.

Besides such interaction disruptor approaches, interference in the movement of the catalytic region of Artemis and DNA-PKcs to prevent Artemis from moving to the DNA end is also an approach that is raised by our results and those of Liu *et al.* (6). In this regard, use of our basal state structure for inhibition of Artemis is an approach that has been successful for allosteric nonreceptor protein tyrosine phosphatase SHP2 inhibition to prevent transition to the activated state (81). Interestingly, SHP2 also contains a disordered C-terminal tail and shows an autoinhibited ‘closed’ conformation, maintained by interdomain interactions (81).

## DATA AVAILABILITY

All the data that support the findings of this study are available from the corresponding authors upon reasonable request.

The cryo-EM maps have been deposited in the Electron Microscopy Data Bank (EMDB): Artemis:DNA-PKcs (EMD-26192, as well as its density modified map and blurred map) and Artemis:DNA-PKcs:DNA (EMD-26198, as well as its blurred map). The coordinates for the Artemis:DNA-PKcs has been deposited in the Protein Data Bank (PDB) under the accession code 7TYR.

## Supplementary Material

gkac564_Supplemental_Files
