# Supplementary material for: Structural analysis of the basal state of the Artemis:DNA-PKcs complex
Source: Nucleic Acids Res. 2022 Jul 8;50(13):7697–720. doi: 10.1093/nar/gkac564 (PMC9303282; doi:10.1093/nar/gkac564)
Supplement: gkac564_Supplemental_Files [file gkac564_supplemental_files.zip › Supplementary_Data.pdf]

Supplementary Data

Structural Analysis of the Basal State of the Artemis:DNA-PKcs Complex

Go Watanabe[1], Michael R. Lieber[1,*], and Dewight R. Williams[2]

[1] Department of Pathology, Department of Biochemistry & Molecular Biology, Department of Molecular Microbiology & Immunology, and Section of Computational & Molecular Biology, USC Norris Comprehensive Cancer Center, University of Southern California Keck School of Medicine, 1441 Eastlake Ave, Rm. 5428, Los Angeles, CA 90089, USA,

[2] Eyring Materials Center, John Cowley Center for High Resolution Electron Microscopy, Arizona State University, Tempe, Arizona 85281, USA

* To whom correspondence should be addressed. Tel: +1 323 865 0568; Fax: +1 323 865 3019 Email: lieber@usc.edu

Supplementary Figure S1. Purification of DNA-PKcs and Artemis and activity assay of Artemis:DNA-PKcs.

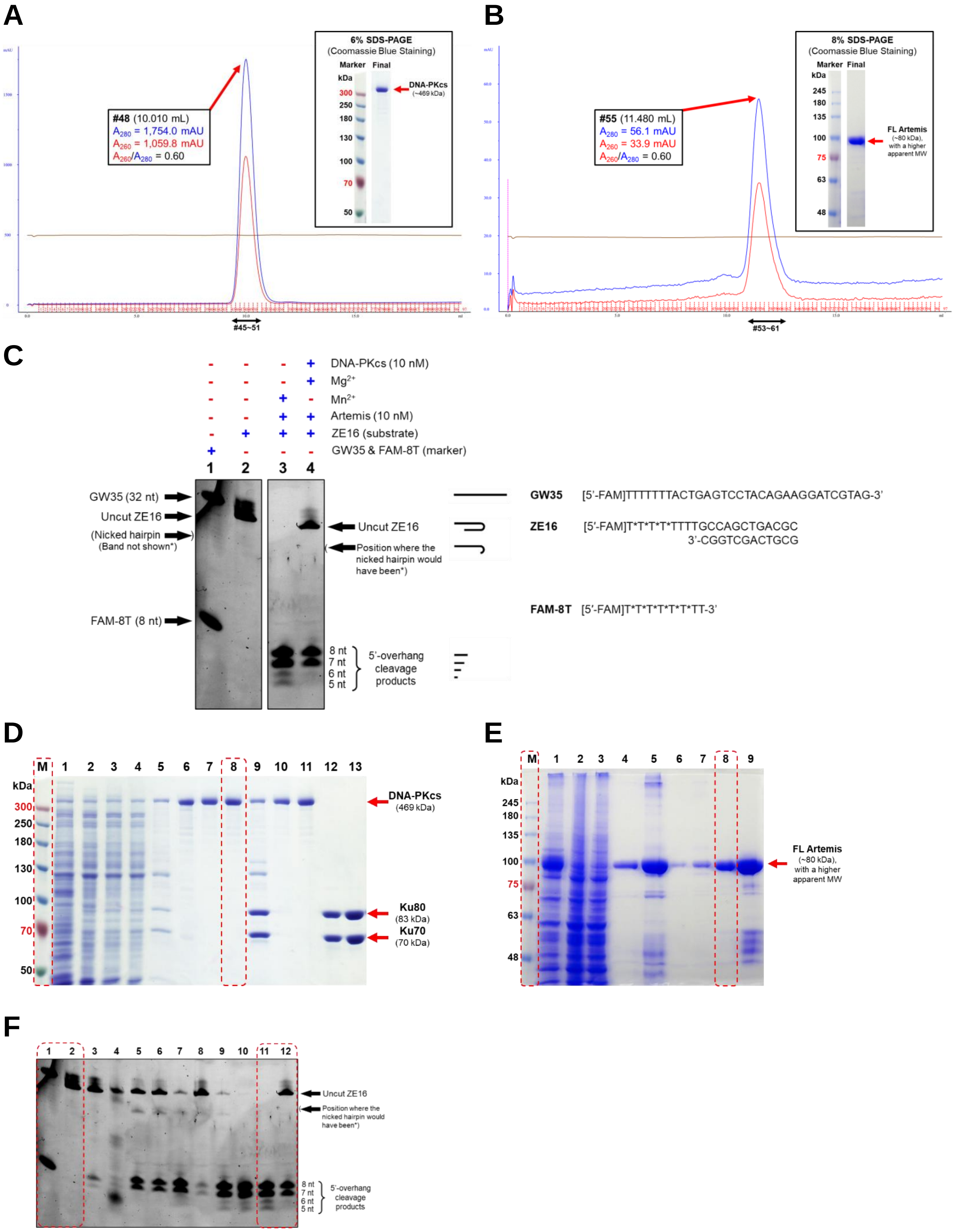

Supplementary Figure S1. Purification of DNA-PKcs and Artemis and activity assay of Artemis:DNA-PKcs. (A) Endogenous DNA-PKcs on a representative Superdex 200 size exclusion chromatography run. The post-Mono S sample was injected onto a Superdex 200 Increase 10/300 GL column pre-equilibrated with a buffer containing 25 mM Tris-HCl (pH 7.5), 200 mM NaCl and 10 mM DTT. The run was monitored with UV at 280 nm (blue line: mAU) and 260 nm (red curved line: axis not shown). The dark red line indicates the conductivity (mS/cm) of the buffer. The active fractions (#45~51), previously detected by *in vitro* kinase assay and SDS-PAGE analysis, were pooled (black arrows). 6% SDS-PAGE was run and the gel was stained with Coomassie Brilliant Blue R250 dye to assess the purity (example shown in a box). The marker and final lanes contained 12 µL of SpectraTM Multicolor High Range Protein Ladder and 1.5 µg of DNA-PKcs, respectively. (B) Recombinant Artemis on a representative Superdex 200 size exclusion chromatography run. The partially purified Artemis was injected onto a Superdex 200 Increase 10/300 GL column pre-equilibrated with a buffer containing 25 mM Tris-HCl (pH 7.5), 200 mM NaCl and 2 mM DTT. The run was monitored as described above. The fractions (#53~61) were pooled. The result of 8% SDS-PAGE gel was shown in a box. The marker and final lanes contained 5 µL of BLUEstain™ Protein Ladder (Gold Biotechnology, St Louis, MO) and ~2 µg of Artemis, respectively. (C) Nuclease assay for Artemis (with Mn^{2+}) and Artemis:DNA-PKcs (ATP/Mg^{2+}). The Artemis substrate, ZE16, was fluorescently labeled at the 5′ end and self-annealed to form an 8-nt 5′ overhang, with the first four nucleotides with phosphorothioate bonds to prevent nuclease degradation, and a 12-bp double-stranded hairpin portion. For the Mn^{2+} assay (lane 3), Artemis without DNA-PKcs was used in the assay condition which contained 10 mM $MnCl_2$ while for the Artemis:DNA-PKcs with ATP/Mg^{2+} assay (lane 4), a pre-formed Artemis:DNA-PKcs complex was used in the assay condition which contained 100 µM ATP and 10 mM $MgCl_2$, instead of $MnCl_2$. The reactions were incubated at 37°C for 30 min and resolved with an 18% denaturing PAGE. The ZE16 nicked hairpin product contained all of the nucleotides of ZE16 except that Artemis nicked 2 nt 3′ of the hairpin tip, and the nucleotides GTCAGCTGGC were no longer present on that product. A 32 nt marker oligonucleotide (GW35) and an 8 nt marker oligonucleotide (FAM-8T) were used to aid gel mobility position. A visual description of substrates and products is shown on the right side of the gel, and the sequences are in Supplementary Table S1. (D) Uncropped gel for Supplementary Figure S1A (Lanes M and 8) (E) Uncropped gel for Supplementary Figure S1B (Lanes M and 8) (F) Uncropped gel for Supplementary Figure S1C (Lanes 1, 2, 11 and 12)

Supplementary Figure S2. Cryo-EM data processing for Artemis:DNA-PKcs.

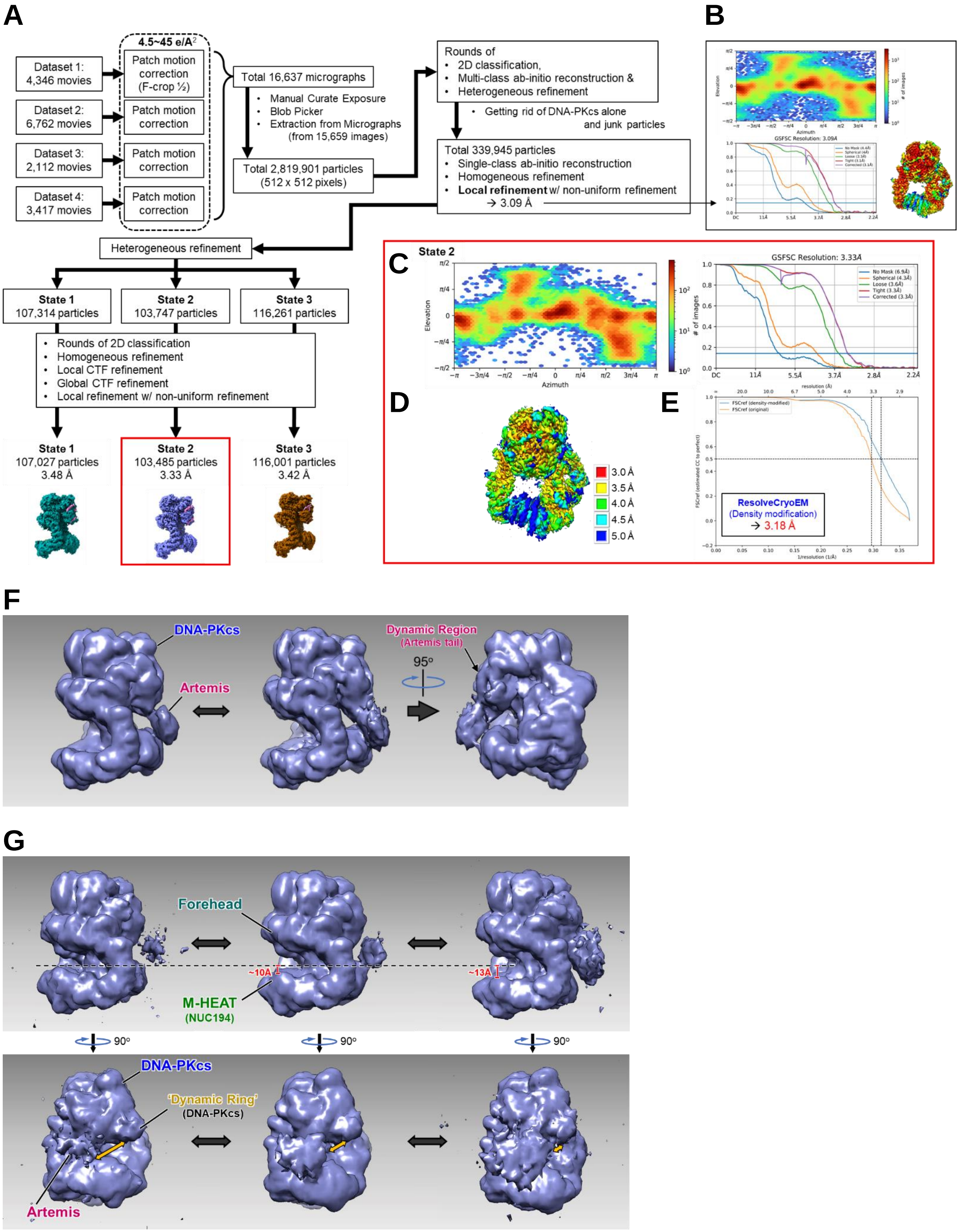

Supplementary Figure S2. Cryo-EM data processing for Artemis:DNA-PKcs.
(A) Cryo-EM data processing for Artemis:DNA-PKcs. Processing was performed in cryoSPARC (v3.2.0+210511). (B) A FSC curve and an angular distribution plot of an intermediate map focusing on Artemis:DNA-PKcs complex (339,945 particles). From this, further heterogeneous refinement was performed. (C) A FSC curve and an angular distribution plot of the state 2 map of Artemis:DNA-PKcs complex (103,485 particles). The resolution was 3.33 Å (FSC at 0.143 cutoff). The half maps were further used for density modification in Phenix. (D) Local resolution analysis of the state 2 map, visualized in Chimera. The color thresholds were set as follows; red: 3.0 Å, yellow: 3.5, green: 4.0, cyan: 4.5 and blue: 5.0. (E) A FSC curve of density modification in Phenix. The resolution was slightly improved to 3.18Å (FSC at 0.5 cutoff). (F) Movie clips from the 3D variable analysis of the state 2 map. The strongest Artemis density was observed among three states. DNA-PKcs and the catalytic domain of Artemis are labeled. The dynamic region of the Artemis tail is pointed. See Supplementary Video 1. (G) Movie clips from the 3D variable analysis of the state 1 map. DNA-PKcs and the catalytic domain of Artemis are labeled. As the forehead and jaw open up (by 10~13Å), the catalytic domain of Artemis appears and the distance between the dynamic ring of DNA-PKcs and the Artemis catalytic domain becomes closer. See Supplementary Video 2.

Supplementary Figure S3. Three different states of Artemis:DNA-PKcs complexes.

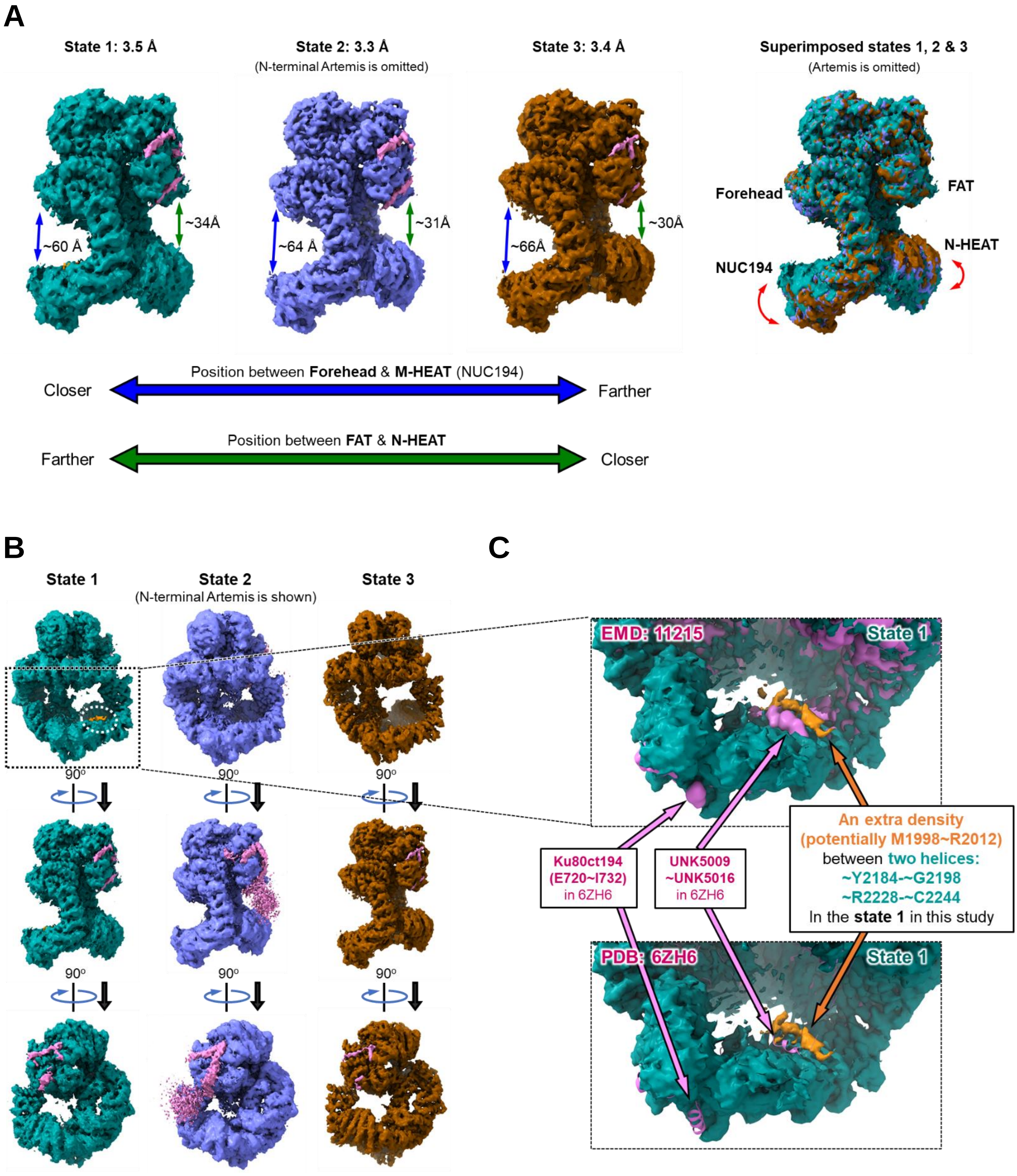

Supplementary Figure S3. Three different states of Artemis:DNA-PKcs complexes.
(A) Comparison of three different Artemis:DNA-PKcs structures which show the dynamic feature of the N-HEAT region and the NUC194 regions of DNA-PKcs. (B) Different views of three states of Artemis:DNA-PKcs structures. An extra density, colored in orange, on DNA-PKcs in the state 1 is circled with white dots. (C) Zoom-in view of the extra density on DNA-PKcs of the state 1 map. This extra density, colored in orange, is potentially a M1998~R2012 region of DNA-PKcs and resides between two helices of M-HEAT (~Y2184-~G2198 and ~R2228-~C2244). Note that this density was not observed in the states 2 and 3 maps. This region was compared with that of the structure of DNA-PKcs:Ku80ct194 (PDB: 6ZH6). In 6ZH6, UNK5009~UNK5016 was assigned in the structure (1). UNK refers to unknown amino acids and are used when those amino acid residues are unidentified, and in Chaplin et al.'s case, poly-ALA was assigned for UNK residues. This could be another mutual exclusive protein-protein interaction site not only with the PQR region but also with other proteins.

Supplementary Figure S4. Comparison of the two newly identified fragments located on the same M-HEAT region in the recently published structures by Liu et al.

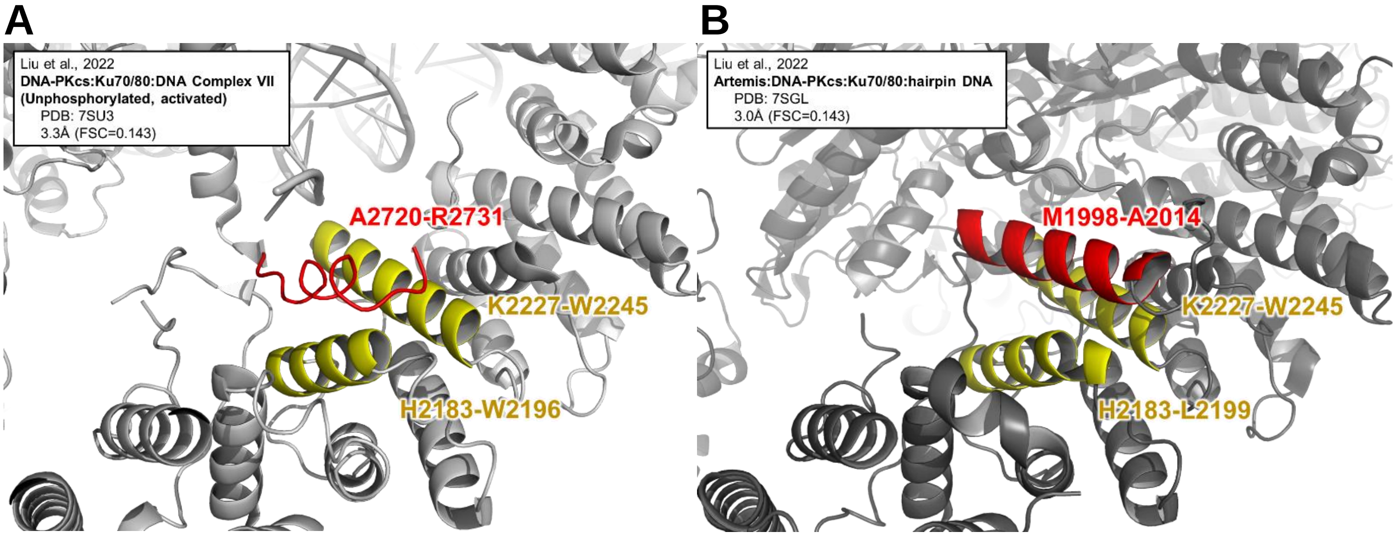

Supplementary Figure S4. Comparison of the two newly identified fragments located on the same M-HEAT region in the recently published structures by Liu et al.
(A) In a recently published structure of DNA-PKcs:Ku70/80:DNA Complex VII (unphosphorylated, activated) (PDB: 7SU3), a fragment of A2720~R2731 (red) was assigned in a density residing on the following two helices (H2183-W2196 and K2227-W2245) (yellow) (2).
(B) In the structure of Artemis:DNA-PKcs:Ku70/80:DNA (PDB: 7SGL), a helix of M1998~A2014 (red) was interacting with the same helices (H2183-L2199 and K2227-W2245) (yellow) (2).

Supplementary Figure S5. Cryo-EM data processing for Artemis:DNA-PKcs:GW132H.

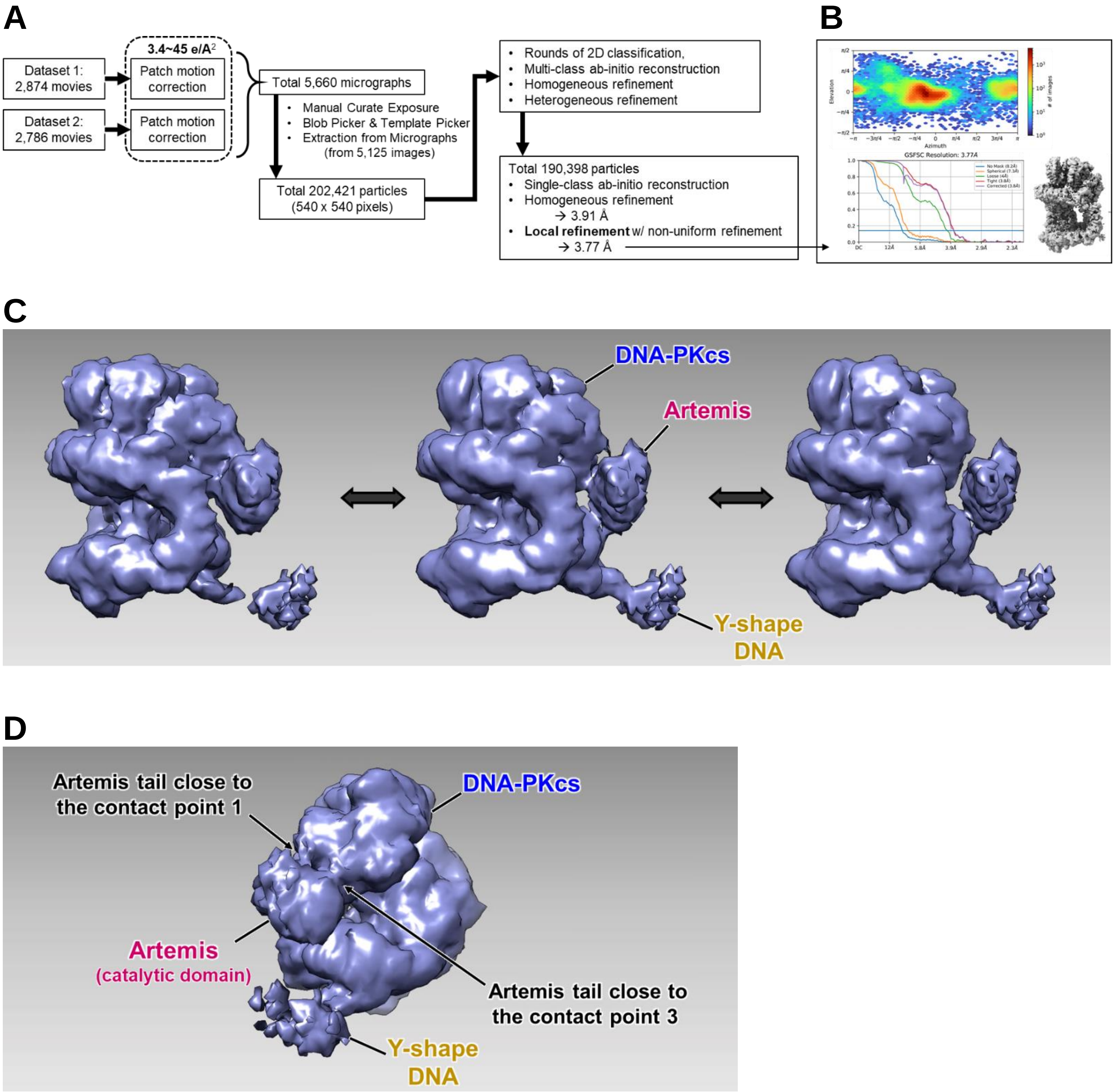

Supplementary Figure S5. Cryo-EM data processing for Artemis:DNA-PKcs:GW132H.
(A) Cryo-EM data processing for Artemis:DNA-PKcs:GW132H. Processing was performed in cryoSPARC (v3.2.0+210511). (B) A FSC curve and an angular distribution plot of an intermediate map focusing on Artemis:DNA-PKcs complex:GW132H (190,398 particles). (C) Movie clips from the 3D variable analysis of Artemis:DNA-PKcs:DNA. The variable position of the Artemis catalytic domain was observed. DNA-PKcs and the catalytic domain of Artemis and Y-shape duplex DNA GW132H are labeled. See Supplementary Video 3. (D) A different view of the movie clips from the 3D variable analysis of Artemis:DNA-PKcs:DNA. A different view of the clips from the panel (C) is shown. The Artemis tail extended from the catalytic domain and located close to the contact point 3 is pointed. Also, the Artemis tail extending from contact point 1 is indicated by an arrow, and this portion may interact with the Artemis catalytic domain.

Supplementary Figure S6. Fitting of the Artemis catalytic region into the blurred map.

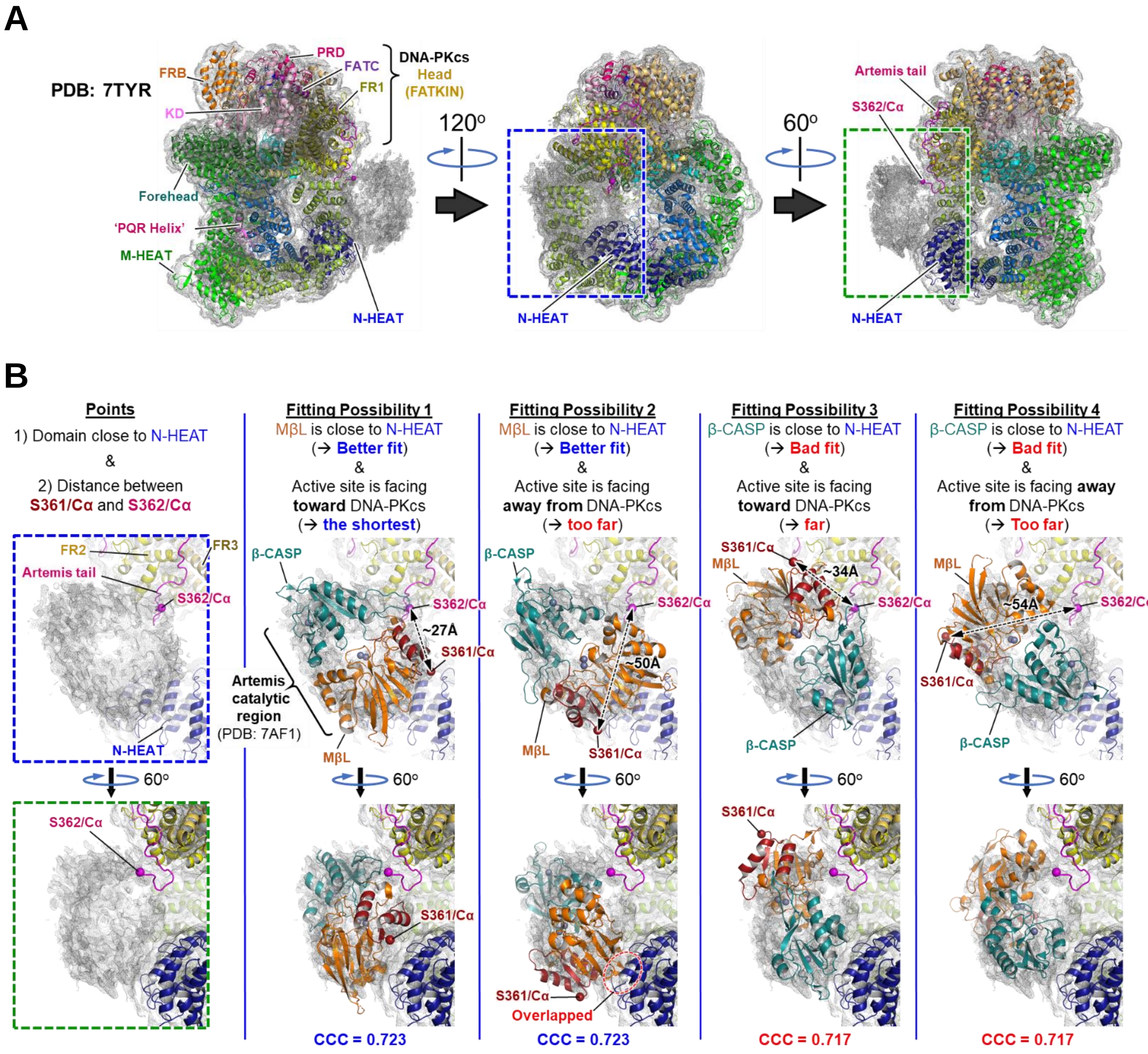

Supplementary Figure S6. Fitting of the Artemis catalytic region into the blurred map.
(A) The blurred map of the Artemis:DNA-PKcs complex and the model of DNA-PKcs and the Artemis tail (PDB: 7TYR). The blue and green boxes correspond to the zoomed areas in the panel B. (B) Four possible fits of the Artemis catalytic region. The crystal structure of Artemis catalytic domain (PDB: 7AF1) was fit into the blurred map. The most probable fit was assessed based on the combination of two choices each: 1) either the MβL domain or the β-CASP domain is close to the N-HEAT repeats of DNA-PKcs, and 2) either the active site of Artemis faces toward or away from DNA-PKcs. Then, the "Fitting Possibility 1 was chosen as the best fit based on two factors: (a) the best match between the triangle shape of the density from the blurred map and the crystal structure of the catalytic domain of Artemis (PDB: 7AF1) and (b) the shortest distance (~27Å gap) between the C-terminal end of the catalytic domain of this crystal structure (e.g., S361) and the N-terminal end of the C-terminal tail (e.g., S362), which was modeled based on our density modified map. Cross-correlation coefficient (CCC) values by Flex-EM were shown at the bottom of the figure. These are speculative fits, and the potential interactions are hypothetical, as shown in Figure 3C. Therefore, the Artemis catalytic region was not included in the deposited model (PDB: 7TYR).

Supplementary Figure S7. cryoDRGN analysis.

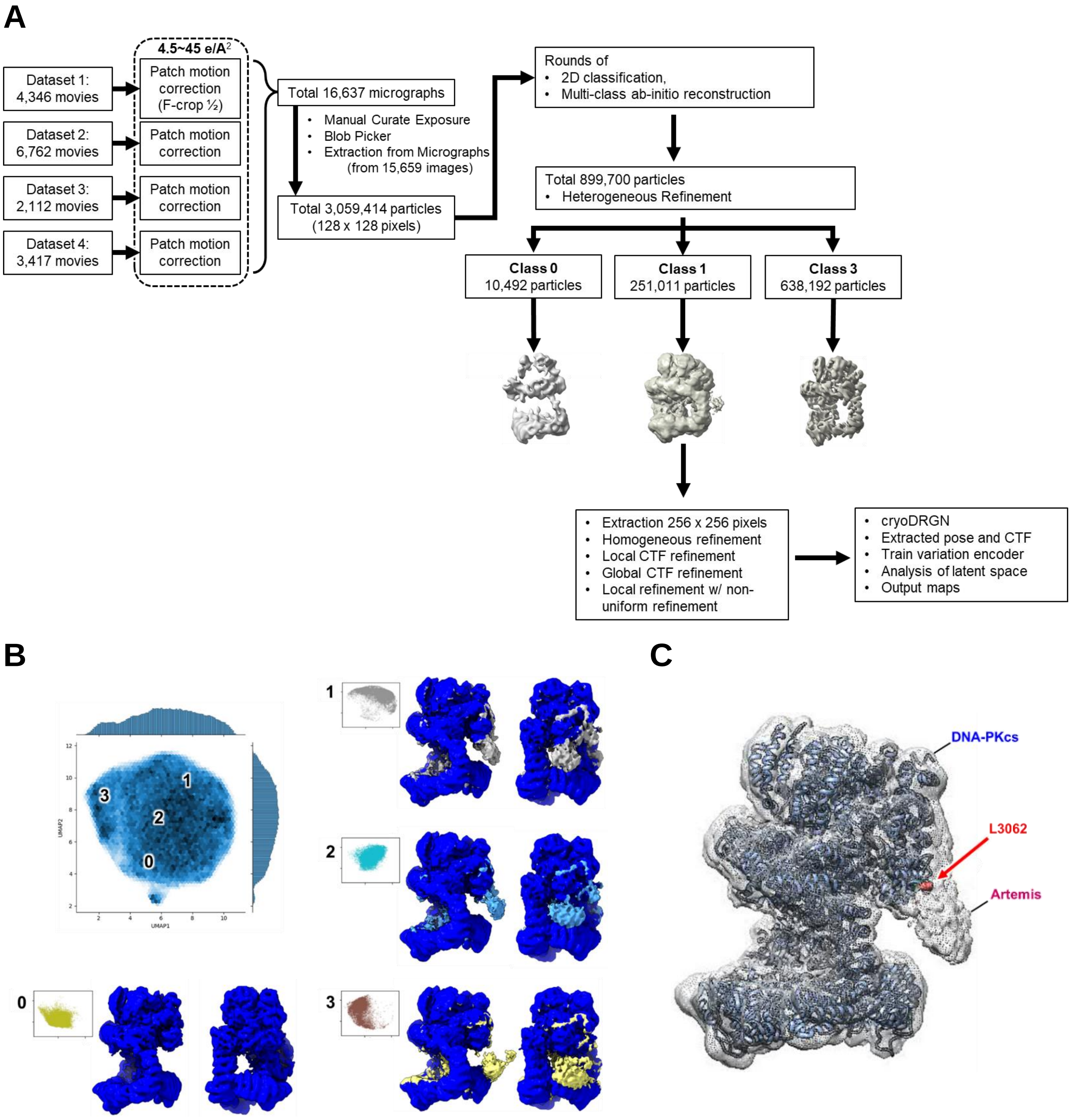

Supplementary Figure S7. cryoDRGN analysis.
(A) Cryo-EM data processing for Artemis:DNA-PKcs for cryoDRGN. (B) UMAP visualization of the latent space representation of particle images along with four select representative density maps of Artemis:DNA-PKcs complex reconstructed by cryoDRGN. The number 0 represents DNA-PKcs alone. A distinct subset of the data contains particles with Artemis which is bound to DNA-PKcs, but it is variable in its binding mode. A full traversal of the UMAP latent space is presented in Supplementary Videos 4, 6 and 7 demonstrates the continuous motions of Artemis catalytic domain relative to DNA-PKcs. (C) One of the Artemis:DNA-PKcs complexes from cryoDRGN analysis with a model. L3062, which is highlighted in red sphere, is located between the FAT domain of DNA-PKcs and the Artemis. See Supplementary Video 5.

Supplementary Figure S8. Interaction between DNA-PKcs and the C-terminal regulatory region of Artemis (basal and activated states).

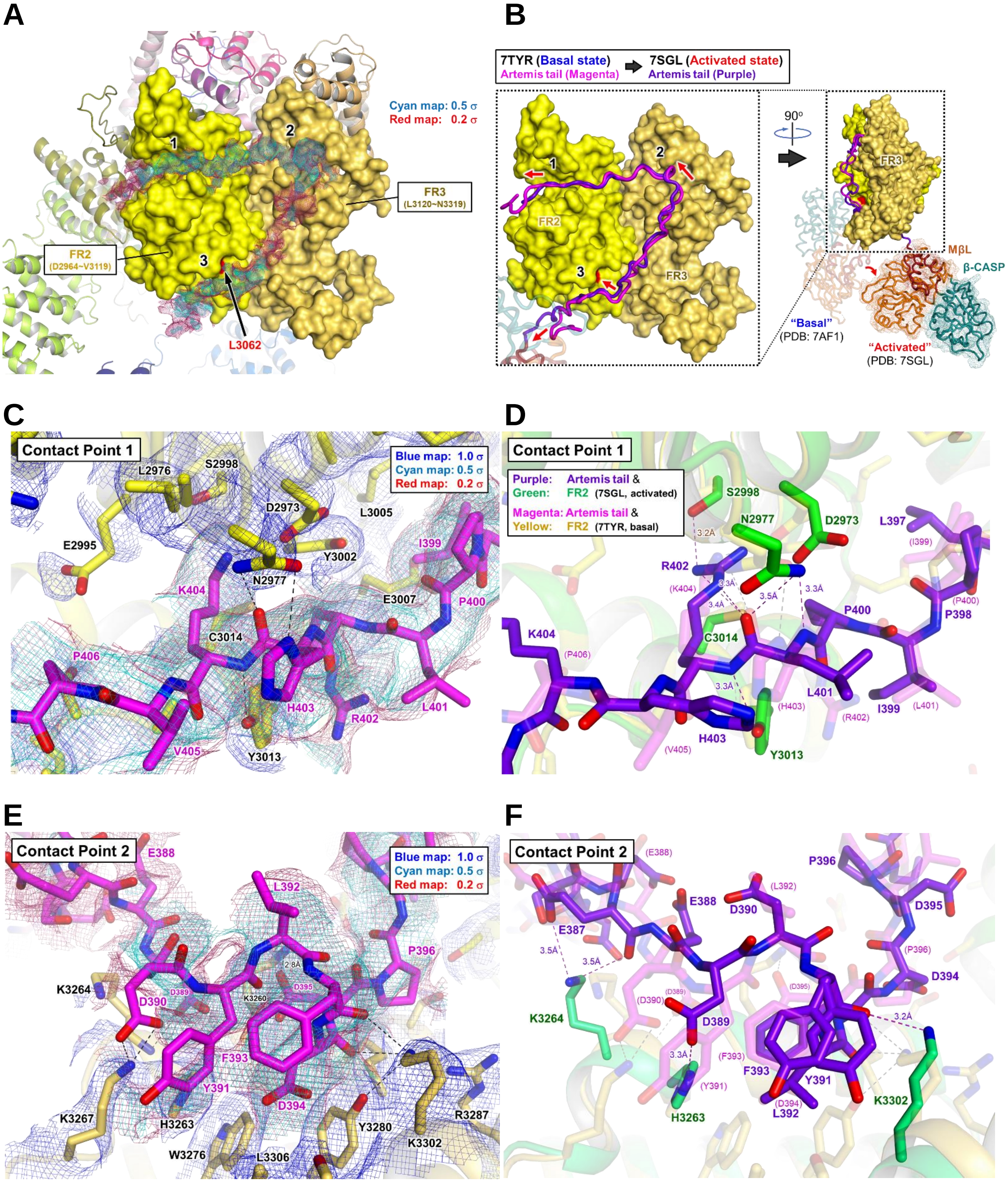

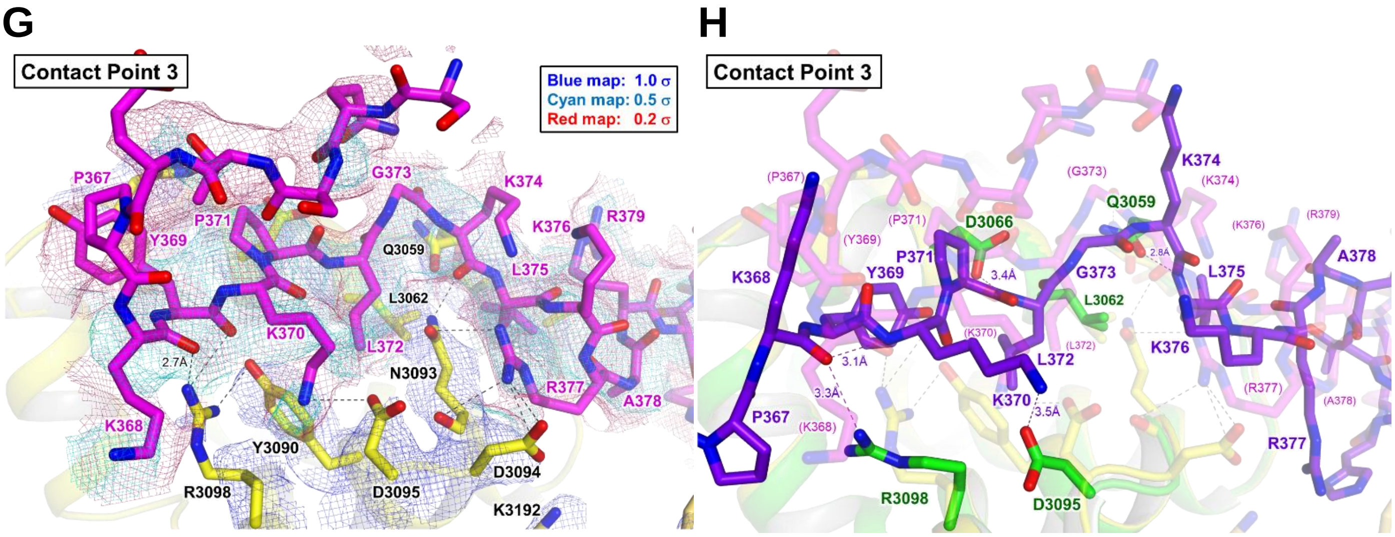

Supplementary Figure S8. Interaction between DNA-PKcs and the C-terminal regulatory region of Artemis (basal and activated states).
(A) The Coulombic potential density of Artemis tail without a model. FR2 (yellow) and FR3 (dark yellow) of DNA-PKcs are shown in surface representation. The density corresponding to the Artemis tail from the density modified map are shown in cyan (0.5 σ) and red (0.2 σ). Three contact points are labeled with numbers. L3062 in FR2 domain is colored in red. (B) Comparison of the Artemis tail between the basal state and the activated state. Our structure (7TYR) and Liu et al.'s structure (7SGL) show similar turn and location on the FAT domain. Upon Artemis activation and relocation to the DNA end, the Artemis tail at contact point 1 shifts to outside of the cleft away from the FR2, and the tail at contact point 2 shifts toward contact point 1, and the tail at contact point 3 moves closer to FR2 to enhance hydrophobic interaction between L372/L375 (Artemis) and L3062 (DNA-PKcs). Red arrows indicate such shifts. (C) Analysis of the interactions in the contact point 1 with the density maps. The Artemis tail is shown in magenta. The density of some potential interacting residues of DNA-PKcs are shown in blue (1.0 σ). (D) Overlaid Artemis tails (basal and activated states) in the contact point 1, highlighting the Activated state. The Artemis tail and the FR2 of the activated state of Artemis:DNA-PKcs:Ku:DNA (PDB: 7SGL) are shown in purple and green, respectively. The Artemis tail and the FR2 of the basal state of Artemis:DNA-PKcs (PDB: 7TYR) are shown in magenta and yellow, respectively. 7TYR is dimmed. (E) Analysis of the interactions in contact point 2 with the density maps. (F) Overlaid Artemis tails (basal and activated states) in the contact point 2, highlighting the Activated state. The color scheme is the same as in the panel C. (G) Analysis of the interactions in contact point 3 with the density maps. (H) Overlaid Artemis tails (basal and activated states) in the contact point 3, highlighting the Activated state. The color scheme is the same as in the panel C. The (C), (E) and (G) correspond to Figures 5B, 5C and 5D, respectively.

Supplementary Figure S9. Analysis of the XRCC4 density in the Art-X4 cleft bound to DNA-PKcs.

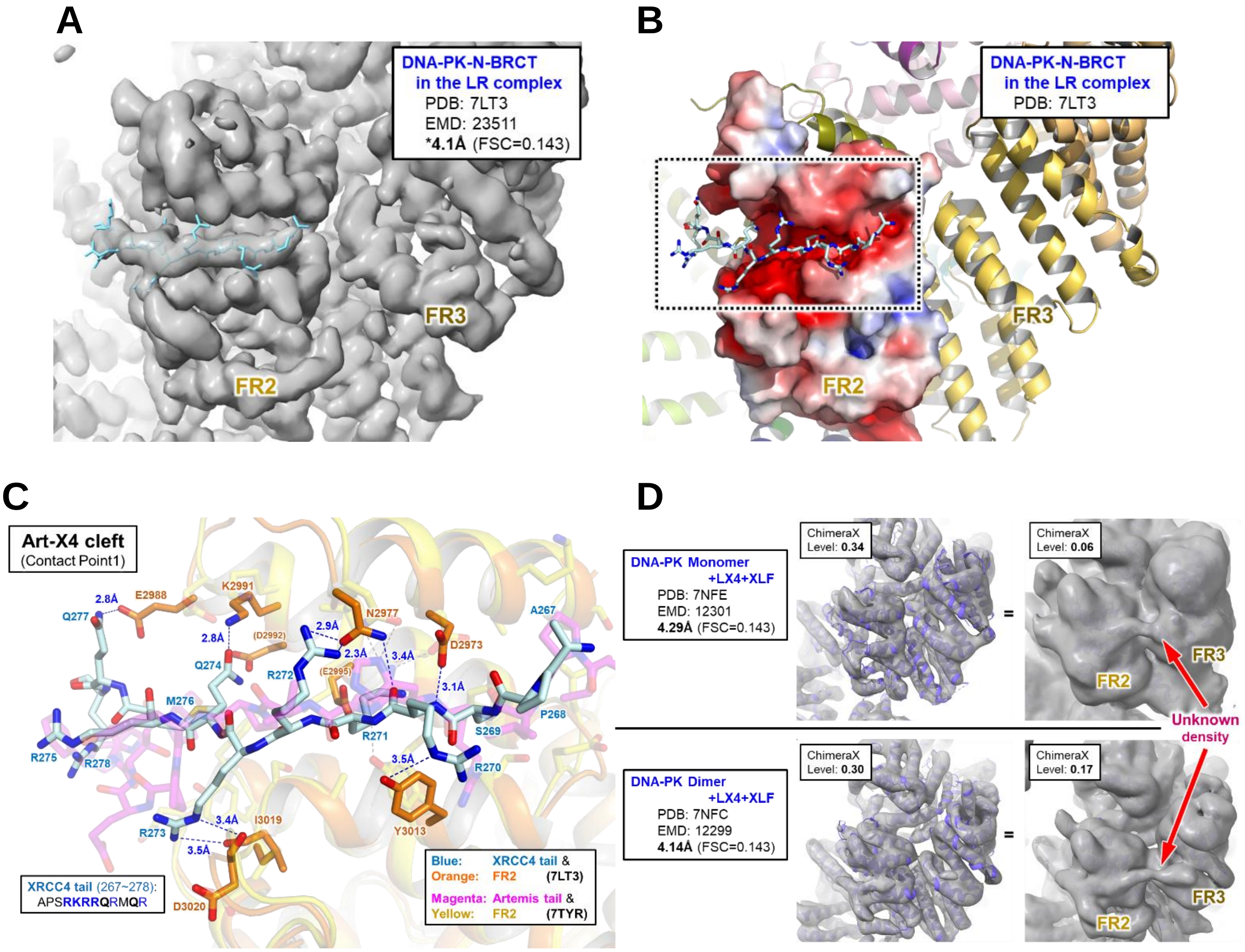

Supplementary Figure S9. Analysis of the XRCC4 density in the Art-X4 cleft bound to DNA-PKcs.
(A) The C-terminal XRCC4 interacting site in DNA-PKcs. In Chen et al.'s 4.60-Å long-range synaptic complex (EMD:23511, later improved to 4.1 Å), a part of the C-terminal tail of XRCC4 (A267-R278) was placed based on the electrostatic surface (PDB: 7LT3) (3). (B) Surface charge distribution of the FR2 subdomain of DNA-PKcs in their structure (PDB: 7LT3). Note that the Art-X4 cleft is a negatively charged region (black dotted box) (3). (C) Analysis of the interaction between the XRCC4 tail and the DNA-PKcs in the Art-X4 cleft. Based on the reported coordinate (PDB: 7LT3), the interaction residues between the XRCC4 tail and the FR2 subdomain of the long-range synaptic complex are analyzed (3). The FR2 and FR3 subdomains of two models (PDBs: 7LT3 and 7TYR) were also aligned to show the overlaid XRCC4 tail and Artemis tail in the Art-X4 cleft. The XRCC4 tail and the FR2 of (7LT3) are shown in blue and orange, respectively. The Artemis tail and the FR2 of the basal state of Artemis:DNA-PKcs (7TYR) are shown in magenta and yellow, respectively. 7TYR and both FR2 cartoons are dimmed. (D) Analysis of DNA-PK monomer+LX4+XLF and DNA-PK dimer+LX+XLF complexes. The maps of the XLF-mediated dimer at 4.14 Å (EMD_12299) and monomer at 4.29 Å (EMD_12301) reported in Chaplin et al. also contain the extra density around the Art-X4 cleft. However, they neither registered any amino acids in this density (PDB: 7NFC and 7NFE) nor discussed the source of this density (4).

Supplementary Figure S10. Full-length Artemis structures predicted by RoseTTaFold and AlphaFold v2.0.

A **RoseTTaFold**

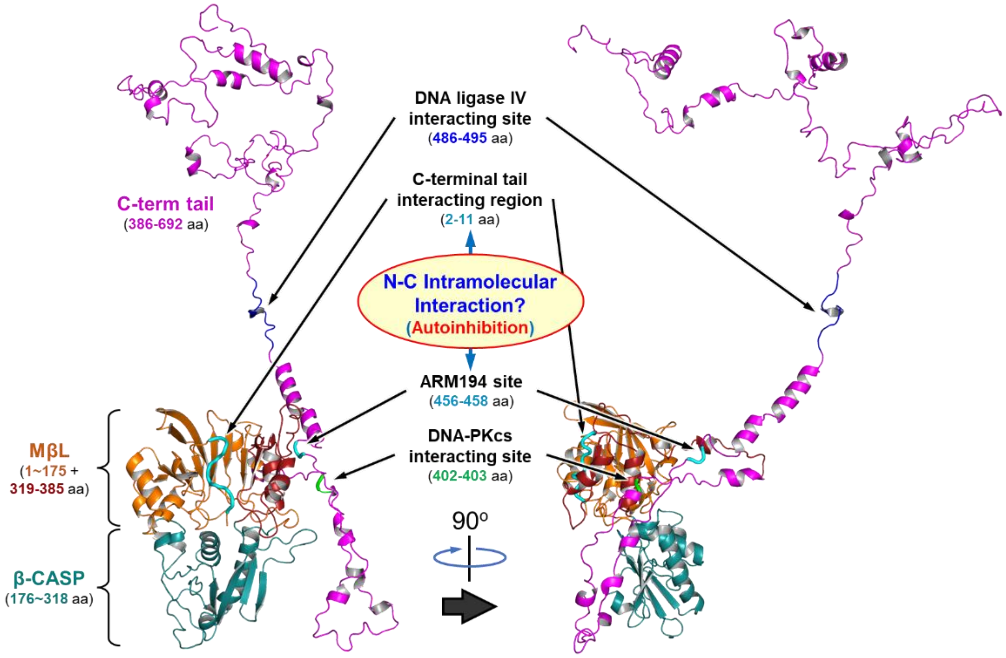

B **AlphaFold v2.0**

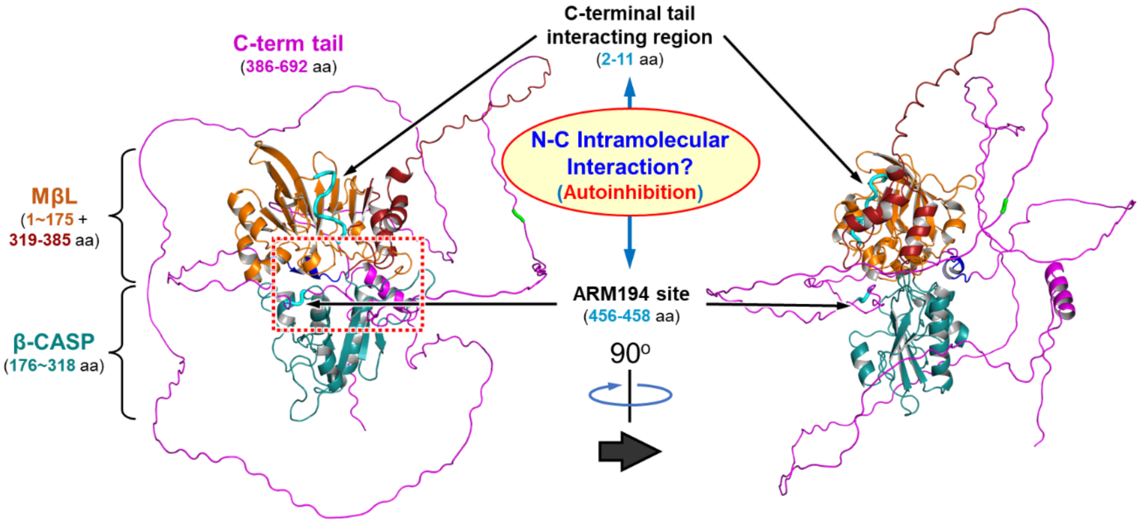

C

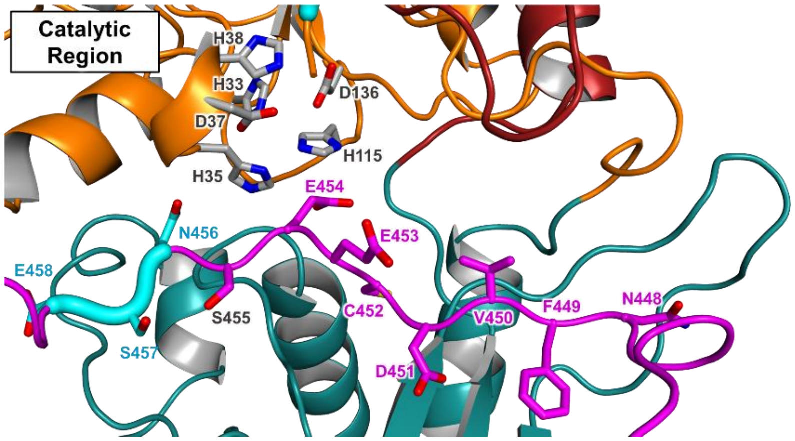

Supplementary Figure S10. Full-length Artemis structures predicted by RoseTTaFold and AlphaFold v2.0. (A) RoseTTaFold analysis on FL-Artemis. The analysis showed an extended C-terminus in all predicted structures, one of which is presented here. The intrinsically disordered C-terminal tail of Artemis is shown in magenta. See Supplementary Video 8. (B) AlphaFold v2.0 analysis on FL-Artemis. The predicted structure is available from the AlphaFold Protein Structure Database (5). The intrinsically disordered C-terminal tail of Artemis is shown in magenta. (C) Zoomed view of the catalytic region of the AlphaFold predicted FL-Artemis. Note that the Artemis tail is located on the surface of the catalytic region.

Table S1. Oligonucleotides used for this study.

**GW1**	[5'-AminoC6]TTTTTAGGCTGTGTTAAGTATCTGCATCGGATCGGGCTCGCCCTCA GAGGACGGCACGTAGTGGC-3'
**GW2**	5'-GCCACTACGTGCCGTCCTCTGAGGGCGAGCCCGATCCGATGCAGATACTTAA CACAGCCT-3'
**GW132**	5'-TTTGGGTGGAGCGCGGGCGTTTTTAGTTTATTGGGCGCGTTTCGCGCCCAGC TTTCCCAGCTAATAAACTAAAAACGCCCGCGCTCCACCCT-3'
**GW35**	[5'-FAM]TTTTTTTACTGAGTCCTACAGAAGGATCGTAG-3'
**FAM-8T**	[5′-FAM]T*T*T*T*T*T*TT-3'
**ZE16**	[5′-FAM]T*T*T*T*TTTTGCCAGCTGACGCGCGTCAGCTGGC-3'

All oligos were purchased from Integrated DNA Technologies, Inc. (San Diego, CA). Asterisks (*) represent phosphorothioate bonds to prevent nuclease degradation.

Table S2. Cryo-EM data collection, refinement and validation statistics.

	**Artemis:DNA-PKcs** EMD-**26192** PDB **7TYR**	**Artemis:DNA-PKcs:DNA** EMDB-**26198**
**Data collection**		
Microscope/Detector	Titan Krios/Gatan K3	Titan Krios/Gatan K3
Magnification	81,000	81,000
Energy filter slit width (eV)	20	20
Voltage (kV)	300	300
Pixel size (Å)	1.08	1.08
Nominal dose (e^-/Å^2)	60	45
Dose rate (e^-/pix/s)	18.5	17.4
Number of frames	40	40
Defocus range (μm)	-0.75 to -3.0	-0.75 to -2.25
**Map reconstitution & refinement**		
Micrographs (used)	12,564	5,660
Used dose (e^-/Å^2)	4.5~45	3.4~45
Symmetry imposed	*C*1	C1
Extraction box size (pixels)	512	540
Initial particles number	2,819,901	202,421
Final particles number	103,747	190,398
Map resolution at FSC = 0.143 (Å) (in cryoSPARC)	3.33	3.77
Map sharpening *B* factor (Å^2)	(+80 for a blurred map*)	(+80 for a blurred map*)
Map resolution at FSC =0.5 (Å) after density modification (by Resolve in Phenix)	3.18*	
**Modeling & refinement**		
Model composition		
Chains	A: DNA-PKcs; C: Artemis tail	
Nonhydrogen atoms	31,610	
Protein residues	3,957	
B factors (Å^2)		
Protein	64.45	
Bonds (RMSD)		
Bond length (Å)	0.005	
Bond angle (°)	1.077	
**Validation**		
MolProbity score	2.13	
Clashscore	8.15	
Rotamer outliers (%)	2.70	
Ramachandran plot		
Favored (%)	94.73	
Allowed (%)	5.10	
Disallowed (%)	0.18	
Rama-Z		
Whole (N = 3948)	0.19	
helix (N = 2507)	1.24	
sheet (N = 43)	1.37	
loop (N = 1398)	-2.04†	
Cβ outliers (%)	0.00	
CaBLAM outliers (%)	3.48	

*The model was built based on a map generated by Resolve (mainly for DNA-PKcs and Artemis tail) (6,7) and a blurred map generated by cryoSPARC (mainly for locating the N-term of Artemis) (8). All refinement statistics were assessed by MolProbity in Phenix dev-4383 (9), wwPDB Validation Service (https://validate.wwpdb.org) and the Quality Control Check v3.2 (https://qc-check.usc.edu).
†Better statistic values were sacrificed to retain some regions that can be observed in the blurred map.

Supplementary Videos

Supplementary Video 1
3D variable analysis (in cryoSPARC) of Artemis:DNA-PKcs State 2

Supplementary Video 2
3D variable analysis (in cryoSPARC) of Artemis:DNA-PKcs State 1

Supplementary Video 3
3D variable analysis (in cryoSPARC) of Artemis:DNA-PKcs:DNA

Supplementary Video 4
cryoDRGN analysis - Dynamic continuous motions of Artemis catalytic domain relative to DNA-PKcs

Supplementary Video 5
cryoDRGN analysis – Second movie

Supplementary Video 6
cryoDRGN analysis – Principal component analysis 1

Supplementary Video 7
cryoDRGN analysis – Principal component analysis 2

Supplementary Video 8
Multiple FL-Artemis models generated by RoseTTAFold

REFERENCES

1. Chaplin, A.K., Hardwick, S.W., Liang, S., Kefala Stavridi, A., Hnizda, A., Cooper, L.R., De Oliveira, T.M., Chirgadze, D.Y. and Blundell, T.L. (2021) Dimers of DNA-PK create a stage for DNA double-strand break repair. *Nat Struct Mol Biol*, **28**, 13-19.
2. Liu, L., Chen, X., Li, J., Wang, H., Buehl, C.J., Goff, N.J., Meek, K., Yang, W. and Gellert, M. (2022) Autophosphorylation transforms DNA-PK from protecting to processing DNA ends. *Molecular cell*, **82**, 177-189 e174.
3. Chen, S., Lee, L., Naila, T., Fishbain, S., Wang, A., Tomkinson, A.E., Lees-Miller, S.P. and He, Y. (2021) Structural basis of long-range to short-range synaptic transition in NHEJ. *Nature*, **593**, 294-298.
4. Chaplin, A.K., Hardwick, S.W., Stavridi, A.K., Buehl, C.J., Goff, N.J., Ropars, V., Liang, S., De Oliveira, T.M., Chirgadze, D.Y., Meek, K. *et al.* (2021) Cryo-EM of NHEJ supercomplexes provides insights into DNA repair. *Molecular cell*, **81**, 3400-3409 e3403.
5. Jumper, J., Evans, R., Pritzel, A., Green, T., Figurnov, M., Ronneberger, O., Tunyasuvunakool, K., Bates, R., Zidek, A., Potapenko, A. *et al.* (2021) Highly accurate protein structure prediction with AlphaFold. *Nature*, **596**, 583-589.
6. Terwilliger, T.C., Ludtke, S.J., Read, R.J., Adams, P.D. and Afonine, P.V. (2020) Improvement of cryo-EM maps by density modification. *Nature methods*, **17**, 923-927.
7. Terwilliger, T.C., Sobolev, O.V., Afonine, P.V., Adams, P.D. and Read, R.J. (2020) Density modification of cryo-EM maps. *Acta Crystallogr D Struct Biol*, **76**, 912-925.
8. Punjani, A., Rubinstein, J.L., Fleet, D.J. and Brubaker, M.A. (2017) cryoSPARC: algorithms for rapid unsupervised cryo-EM structure determination. *Nature methods*, **14**, 290-296.
9. Liebschner, D., Afonine, P.V., Baker, M.L., Bunkoczi, G., Chen, V.B., Croll, T.I., Hintze, B., Hung, L.W., Jain, S., McCoy, A.J. *et al.* (2019) Macromolecular structure determination using X-rays, neutrons and electrons: recent developments in Phenix. *Acta Crystallogr D Struct Biol*, **75**, 861-877.
